# Choosing depression treatment in type 2 diabetes: integrating psychiatric efficacy, metabolic effects, and cardiovascular risk. A narrative review

**DOI:** 10.3389/fpsyt.2026.1976656

**Published:** 2026-10-19

**Authors:** Yanyan Tan, Yuchen Zhang, Lang Yuan, Fei Huang, Yanbin Cheng, Feng Cheng

**Affiliations:** 1 Clinical Laboratory, Ezhou Central Hospital, Ezhou, China; 2 School of Laboratory Medicine, Hubei University of Chinese Medicine, Wuhan, China; 3 School of Health and Nursing, Wuchang University of Technology, Wuhan, China

**Keywords:** antidepressants, cardiometabolic risk, collaborative care, depression, psychotherapy, shared decision-making, type 2 diabetes

## Abstract

Depression in people with type 2 diabetes (T2D) complicates daily self-management and treatment selection. Psychiatric benefit must be considered alongside weight, glycaemia, cardiovascular tolerability and interactions. This narrative review draws on purposively selected literature and distinguishes direct T2D evidence from mixed-diabetes studies, observational associations and indirect evidence. Study selection and extraction were not consistently duplicated, and no formal *de novo* risk-of-bias or GRADE assessment was undertaken. A revision-stage qualitative appraisal makes study limitations and population differences explicit. The main T2D pharmacotherapy network comprised 12 short-term trials with 792 participants; its sparse comparisons do not establish a reliable prescribing hierarchy. Individual-agent data suggest clinically relevant differences, but favourable mean HbA1c changes do not establish freedom from hypoglycaemia or hyperglycaemia. Psychological, exercise, digital and collaborative-care interventions also differ in diagnostic eligibility, support intensity and metabolic findings. Cardiovascular tolerability, intermediate safety markers and prevention of clinical events require separate interpretation. Cardiac studies cannot establish T2D-specific cardioprotection. We propose an unvalidated conceptual checklist in which psychiatric urgency precedes cardiometabolic constraints, prior response and patient preferences. Monitoring examples are extrapolated from guidance and pharmacological safety information, not tested T2D-specific schedules. The checklist is intended to structure discussion, not to function as a guideline, validated decision aid or prediction rule.

## 1 Introduction

Depression occurs considerably more often in people with diabetes. In 44 controlled studies, its prevalence was 19% among people with diabetes and 11% among those without diabetes (odds ratio 1.76) (1). Depressive symptoms can interfere with sleep, activity, diet, medication use and glucose monitoring. In the other direction, treatment burden, diabetes distress, hypoglycaemia and complications may sustain low mood. In T2D, depression is associated with both microvascular and macrovascular complications, including cardiovascular events (2, 3). Its association with mortality points to a more vulnerable population, but observational studies cannot establish that treating depression prevents vascular events (4).

Psychiatric and diabetes guidelines address many of the relevant issues, usually in separate sections, and give little direction on a combined treatment hierarchy. Diabetes guidance includes emotional well-being. Depression guidance selects psychological treatment, medication or both according to severity, prior response, safety, comorbidity and patient preference (5, 6). Trials in major depressive disorder provide estimates of average antidepressant efficacy, but those estimates cannot simply be carried over to T2D (7). The balance between otherwise plausible options may change in the presence of obesity, poor glycaemic control, cardiovascular disease, arrhythmia susceptibility, kidney or liver dysfunction, autonomic symptoms, antithrombotic therapy or polypharmacy.

Studies conducted specifically in T2D remain scattered. The main pharmacotherapy network included 12 trials, only 792 participants and follow-up of 8 to 24 weeks, so most comparisons were sparse (8). A broader review and a later network meta-analysis of non-pharmacological treatments found improvement in depression, but glycaemic findings were inconsistent (9, 10). Collaborative-care trials improved psychiatric and medical outcomes at the same time, without showing how much benefit came from any individual antidepressant (11, 12). There is more evidence on SSRI-related glycaemic change, antidepressant-associated weight change and other cardiometabolic outcomes, although much of it is short term. Trials in related cardiovascular populations rarely establish effects on clinical events (13–19).

This review examines how evidence provenance and follow-up affect the interpretation of treatment findings. We distinguish drug effects from changes associated with depression recovery or coordinated care, and psychiatric outcomes from cardiometabolic surrogates and clinical events. The contribution is an interpretive synthesis and an author-proposed checklist, not a comprehensive evidence map or a new treatment hierarchy (20).

The reciprocal behavioural and biological pathways linking depression and T2D, and their implications for treatment choice, are summarised in **Figure 1**.

**Figure 1:**
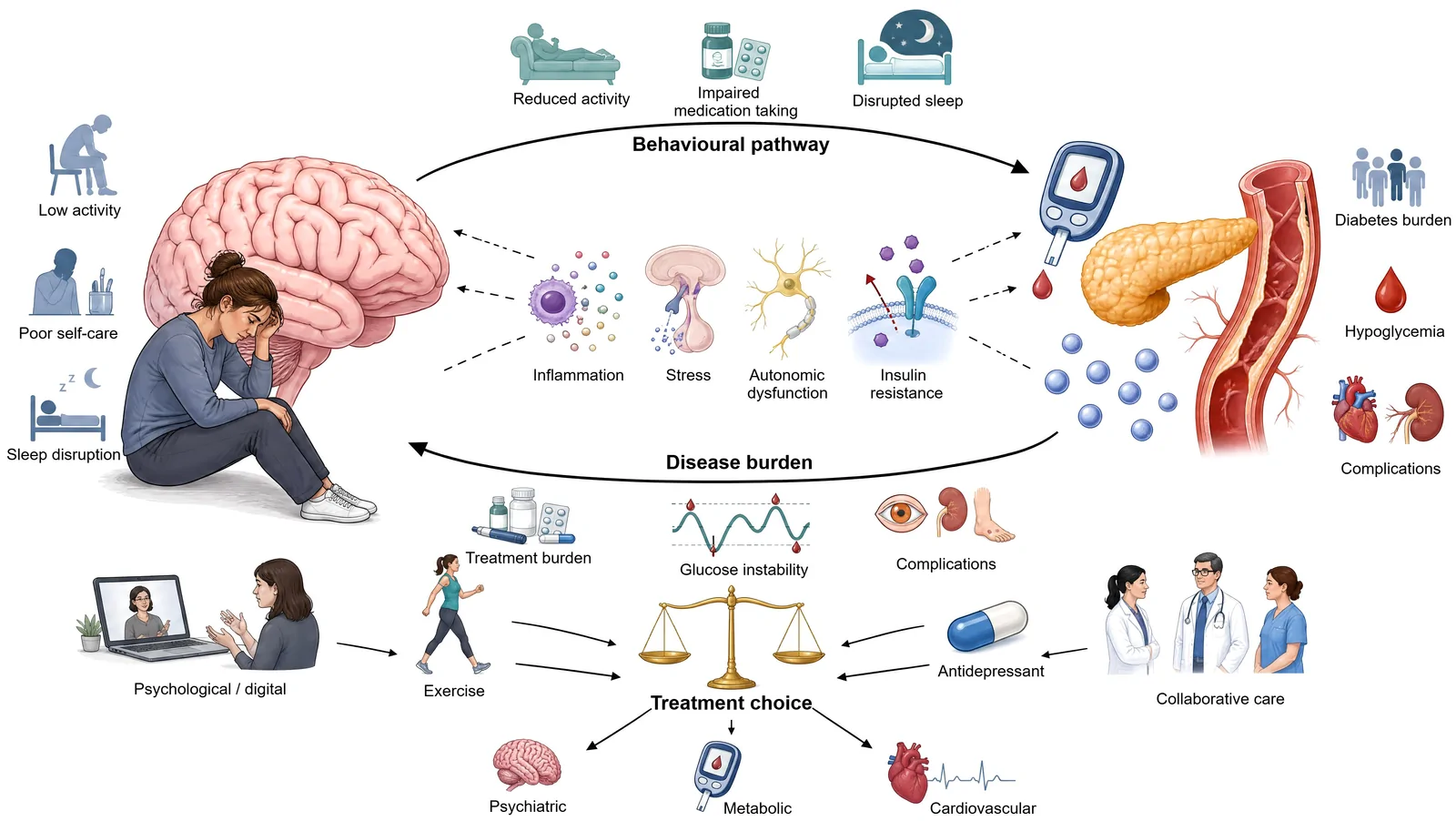
Bidirectional pathways linking depression, type 2 diabetes and treatment choice. The upper and lower solid arrows summarise behavioural and disease-burden associations, respectively; they do not establish causal effects in either direction. The central dashed arrows indicate proposed biological links involving inflammation, stress-system activation, autonomic dysfunction and insulin resistance, drawing on mechanistic and observational evidence rather than direct comparisons of depression treatments in T2D. The lower treatment-choice schematic is the authors’ conceptual synthesis. Selected T2D trials inform psychiatric and some metabolic outcomes, whereas much cardiovascular and drug-safety information is extrapolated from other populations (**Tables 1**, **2**; **Supplementary Tables S2**, **S3**). Neither arrow style nor icon size grades certainty or establishes a treatment hierarchy. T2D, type 2 diabetes.

## 2 Review scope and methods

### 2.1 Review design, scope and eligibility

This is a narrative review of purposively selected literature, not a systematic or scoping review. It was not prospectively registered and does not claim exhaustive study identification. PRISMA-ScR and SWiM are cited to distinguish their intended reporting settings, not to imply compliance (21, 22). The clinical tables and figures are conceptual aids to interpretation rather than systematically derived recommendations.

The clinical focus is adults with T2D and a depressive disorder or elevated depressive symptoms. We distinguish diagnostically established major depressive disorder (MDD), other or subthreshold depressive disorders, elevated scores without diagnostic confirmation, and diabetes distress without a depressive disorder. The last category informs diagnostic triage, not evidence for antidepressant treatment. Mixed or unspecified diabetes populations are partially direct unless T2D results are separately reported. Neuropathic-pain studies without depression, general-MDD studies and cardiac trials inform only the outcomes relevant to those populations. Treatments considered include medication, psychotherapy, exercise, digitally delivered treatment, collaborative care and combinations.

### 2.2 Literature selection and revision-stage source verification

Literature selection was purposive, organised around depression treatment in T2D, metabolic effects, cardiovascular and other safety outcomes, and care delivery. The original review used a literature cutoff of 27 July 2026, but the original search documentation was unavailable for verification during revision, preventing reconstruction of complete database-specific searches, dates, restrictions or screening decisions. We therefore do not present the original search as an exhaustive six-database search. The PubMed expression retained from the original manuscript is reproduced in **Supplementary Table S1** as an example, not a complete search strategy. Missing search histories, deduplicated record counts and exclusion reasons have not been retrospectively reconstructed.

During revision, selected cited studies, reviewer-suggested publications and regulatory safety sources were checked through PubMed-indexed records, DOI/publisher records and available full text. **Supplementary Table S1** distinguishes this targeted verification from the original search and records source-access limits. Selection and extraction were not consistently independently duplicated; no reproducible historical disagreement-resolution process can be reported. The source inventory identifies each cited report and its role. Related reports were linked where identified, including the bupropion cohort and its sexual-function analysis, and the acute and follow-up reports of Program ACTIVE II. These are not independent replications. Review-level syntheses also overlap with individual studies.

### 2.3 Evidence appraisal, synthesis and transferability

No formal *de novo* RoB 2, ROBINS-I, ROBINS-E, AMSTAR 2 or GRADE assessment was performed (23–27). **Supplementary Table S2** provides a structured qualitative appraisal of selected studies that materially inform the comparisons. Fields include design and sample size, diabetes population, depression ascertainment, intervention and comparator, follow-up, psychiatric and cardiometabolic outcomes, major limitations, directness, and funding or conflicts where verifiable. Abstract-only retrieval and unverified disclosures are labelled. These judgements are not validated quality scores, certainty ratings or a substitute for independent full-text risk-of-bias assessment.

Provenance is assigned to the claim and outcome, not simply to the paper. Direct evidence requires T2D plus a stated depressive diagnosis or symptom threshold; these populations remain separately labelled. Mixed-diabetes studies without a T2D estimate are partially direct. General-depression trials supply bridge efficacy evidence, whereas cardiac trials and regulatory information supply bridge safety evidence for the outcomes examined. Mechanistic reports, case reports and expert consensus provide context. A study may contribute to several domains. An absent T2D-specific analysis, a pain sample without depression, or a surrogate substituted for clinical events is a major mismatch for the corresponding efficacy claim. **Supplementary Table S3** defines these rules with examples. Categories were applied descriptively during revision, without independent agreement testing. They constrain wording to population-specific findings, conditional extrapolation or hypotheses rather than graded recommendations.

The evidence-transfer framework used to distinguish direct T2D studies from bridge and safety evidence is shown in **Figure 2**. Its legend explains the source categories and the limits of transferring findings between populations and outcomes.

**Figure 2:**
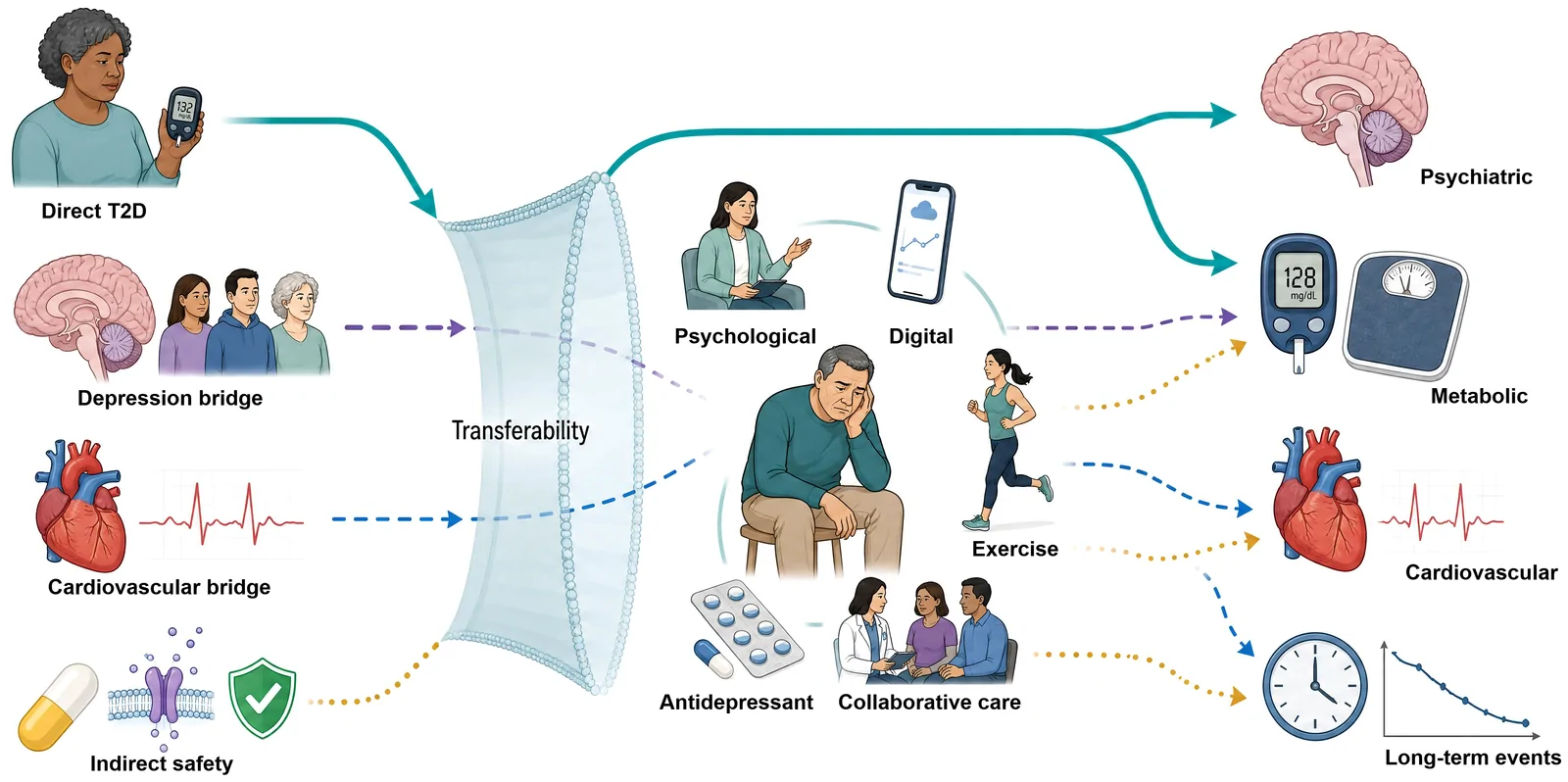
Evidence provenance and transferability across psychiatric, metabolic and cardiovascular outcomes. The teal solid pathway represents direct T2D evidence; purple dashed and blue dashed pathways represent extrapolation from depression and cardiovascular populations, respectively; the orange dotted pathway represents indirect pharmacological or safety information. These source categories are not certainty grades. The transferability filter is an author-proposed interpretive step, not a validated assessment instrument. Applicability depends on the study population, depression ascertainment, intervention, comparator, outcome and follow-up; mixed-diabetes findings without T2D-specific estimates are only partially direct. The outcome icons identify domains for assessment, not benefits established for every treatment. In particular, pathways towards long-term events do not imply proven cardiovascular prevention in T2D, and the small descending curve is schematic, not an observed treatment effect. Claim-specific limitations are detailed in **Supplementary Tables S2**, **S3**. T2D, type 2 diabetes.

## 3 Interpretive principles for multidimensional treatment selection

Depressive disorder, elevated depressive symptoms and diabetes distress are related but not interchangeable. Distress may arise from the demands of diabetes care without meeting MDD criteria, while a positive symptom screen requires diagnostic assessment. Distress and depression are associated with poorer diabetes self-care, but neither association establishes an antidepressant indication for distress alone (2–4, 28, 29). The study appraisal separately records diagnostic interviews, other diagnostic criteria and symptom thresholds when available.

Cardiometabolic outcomes can change for several reasons. The drug may alter appetite, weight, glucose, lipids, blood pressure, heart rate or cardiac conduction. At the same time, relief of depression may improve sleep, activity, adherence and self-management. Collaborative care adds another pathway by changing access, measurement and treatment adjustment. Most trials observe these processes together and do not randomise or formally model the mediators. For that reason, an HbA1c change during antidepressant or collaborative-care treatment cannot be attributed to the drug without additional mechanistic or mediation evidence (11–15, 30).

For this discussion, acute outcomes refer approximately to the first 12 weeks, continuation to the subsequent period through 12 months, and longer-term outcomes to follow-up beyond 12 months. These are review conventions, not prescribed treatment durations. Response generally denotes substantial symptom reduction, often at least 50%; remission denotes a low symptom level or loss of diagnostic criteria. Relapse is return of illness before sustained recovery, whereas recurrence is a new episode after recovery. Study-specific thresholds and time requirements vary and are retained rather than treated as interchangeable. Antidepressant adherence, diabetes-medication adherence and broader self-management are also distinguished.

Most T2D depression trials cannot determine long-term cardiovascular events, complications or mortality (8, 13–19). An HbA1c, weight or blood-pressure change may be relevant without demonstrating fewer events. This distinction governs the comparisons and the unvalidated checklist in Section 7.

## 4 Psychiatric efficacy and acceptability of depression treatments in T2D

### 4.1 Psychological, digital, exercise and behavioural interventions

Psychological interventions can reduce depression in T2D, but populations and treatment components differ (31, 32). The original CBT trial enrolled T2D with major depression, whereas CBT for adherence and depression (CBT-AD) included current major depressive episodes, dysthymia and partially remitted MDD (33, 34). CBT-AD explicitly addressed diabetes-medication use and glucose monitoring, so its HbA1c and adherence findings cannot be assigned solely to mood improvement. The DAD comparison of CBT and sertraline enrolled mixed diabetes types and supplies partially direct evidence (35). **Supplementary Table S2** records these distinctions.

Digital delivery is not a single intervention. Therapist-supported web CBT in mixed diabetes with diagnosed MDD and guided web self-help in symptom-selected mixed diabetes samples reported benefit (36, 37). In contrast, unguided SpringboarD in T2D with depressive symptoms did not show a specific advantage over its active control (38). Asynchronous web modules with feedback differ from synchronous telephone counselling, which may incorporate walking support (39). Exercise and behavioural programmes can improve mood and function, but glycaemic findings vary; favourable baseline-HbA1c subgroup findings in Program ACTIVE II remain exploratory (40–42). Its acute and follow-up reports concern the same trial. These interventions do not replace urgent psychiatric care for immediate risk or severe impairment.

### 4.2 Antidepressant pharmacotherapy

General-MDD trials support antidepressant efficacy, but direct comparisons in T2D remain sparse (7, 8). The T2D network included 12 trials with 792 participants followed for 8–24 weeks. Its large estimates for some agents arise from a small network and are not a stable ranking or evidence of established long-term safety. Duloxetine and bupropion were not included in that network. **Table 1** and **Supplementary Table S2** distinguish agent-specific findings from class-level assumptions and separate average glycaemic change from reported hypoglycaemia or hyperglycaemia.

**Table 1:** Agent-level evidence and safety considerations for depression treatment in T2D.

Agent	Psychiatric evidence	Glycaemia and weight	Cardiovascular/other safety considerations	Provenance and principal limitation
Sertraline	Acute diabetes studies vary; recurrence prevention shown after prior response.	Variable HbA1c findings; no maintenance-phase HbA1c advantage. Hypo-/hyperglycaemia reports do not quantify risk. Weight can change.	Bleeding warning; QTc caution with risk factors; hyponatraemia and withdrawal possible. Hepatic dose constraints; review interactions.	D/P: (35, 43, 44). B: (14, 16, 95). Mixed diabetes and responder-enriched maintenance limit generalisation.
Escitalopram	Favourable short-term findings in a sparse T2D network and small trial.	Small-study HbA1c signal; hypo-/hyperglycaemia reported postmarketing. Modest relative weight gain in non-T2D cohort.	Dose/exposure-related QTc concern; bleeding and hyponatraemia warnings. Consider hepatic impairment and severe renal uncertainty.	D: (8, 46). B: (14, 17, 47). Positive ACS event trial is not T2D-specific evidence.
Fluoxetine	Improved symptoms in mixed diabetes, only 34/60 participants with T2D.	HbA1c advantage not significant in trial. Label reports hypoglycaemia during treatment and hyperglycaemia after withdrawal. Early appetite/weight loss can occur.	Bleeding/QTc warnings; CYP2D6 inhibition and long half-life complicate interactions and switching. Hepatic impairment affects exposure.	P: (45). B: (14, 84, 85, 96). Short mixed-diabetes study; no durable metabolic superiority.
Duloxetine	General-MDD efficacy; neuropathic-pain efficacy is a separate indication.	DPNP studies show modest glucose increase; non-DPNP analysis largely neutral. Short-term weight loss can give way to gain.	BP elevation, orthostasis and bleeding; hepatic disease and severe renal impairment constrain use. CYP2D6 interactions; discontinuation symptoms.	B: (7, 50–52). DPNP is not depression-selected evidence; no T2D efficacy ranking.
Bupropion	Improvement in an open-label T2D cohort; secondary sexual-function analysis.	HbA1c and BMI declined without a control arm. Both directions of dysglycaemia reported; lower mean weight gain in non-T2D cohort.	Hypertension and dose-related seizures; hypoglycaemia can increase seizure susceptibility. CYP2D6 inhibition; renal/hepatic dose review.	D observational: (48, 49). B: (14, 53). Sexual-function report shares parent cohort; no causal glucose-lowering claim.
Agomelatine	Small active-comparator T2D studies with comparator-dependent results.	Endpoint HbA1c findings vary; event-level dysglycaemia poorly characterised. No consistent weight advantage; label lists weight increase.	Principal concern is hepatotoxicity. Baseline and scheduled liver tests required; liver disease and strong CYP1A2 inhibitors restrict use.	D: (46, 54–56). B: (57). Small, brief studies and reporting weaknesses; no established cardiac-event benefit.
Vortioxetine	T2D pilot against sertraline; contextual pharmacology review.	Exploratory favourable metabolic changes; only 21/50 completed. Little mean weight change in general trials does not mean no risk.	Limited average QT effect in thorough-QT study; bleeding warning remains. Strong CYP2D6 inhibitors require dose review; sexual effects can occur.	D: (59). C: (58). B: (60). Severe attrition and short follow-up; no T2D glycaemic or event superiority.
Trazodone	Expert consensus supports selected MDD use; low-dose sleep treatment is not equivalent to antidepressant dosing.	No T2D comparative glycaemic benefit established by cited consensus. Weight gain and loss appear in labelling.	Sedation, falls, orthostasis, QT prolongation and bleeding; CYP3A4 interactions. Renal/hepatic impairment lacks adequate study.	C: (61). B: (62). Delphi opinion, not patient-level diabetes trial; cannot infer low cardiac or bleeding risk.

D, direct T2D; P, partially direct mixed diabetes; B, bridge efficacy/safety, guidance or product information; C, contextual expert opinion. Provenance is not certainty. All adverse-event cautions in the safety column derive from non-T2D regulatory or bridge evidence unless otherwise stated. No agent is ranked as best. DPNP, diabetic peripheral neuropathic pain; ACS, acute coronary syndrome; MDD, major depressive disorder. Local product information governs indications, contraindications and dosing.

Sertraline has evidence for acute treatment and recurrence prevention in diabetes, but glycaemic findings vary (35, 43, 44). A maintenance trial randomised 152 patients who had recovered during open-label sertraline; 82.9% had T2D. Continued sertraline reduced recurrence, while HbA1c did not differ from placebo in the randomised phase (43). Fluoxetine improved depressive symptoms in an eight-week trial of 60 participants, only 34 of whom had T2D, without a significant glycated-haemoglobin advantage (45). Escitalopram has favourable short-term findings in the sparse network and a small open-label comparison, but these require confirmation and must be considered alongside its QTc liability (8, 46, 47).

Bupropion was associated with improvement in depression, BMI and HbA1c during an uncontrolled T2D cohort study (48). A secondary analysis of that cohort reported improved sexual functioning; it is not an independent controlled trial (49). Recovery, self-management and weight change may contribute to these observations. Duloxetine may be relevant when depression coexists with painful neuropathy, but diabetic neuropathic-pain trials recorded modest fasting-glucose increases and a greater long-term HbA1c rise than routine care (50). A largely glycaemically neutral meta-analysis of other indications is indirect and does not negate those findings (51). Blood pressure, organ function and interactions remain relevant (52, 53).

Agomelatine has been examined in several small active-comparator T2D studies. A four-month open-label pilot involving 40 patients favoured agomelatine over sertraline for symptoms and endpoint HbA1c (54). A 12-week randomised comparison involving 116 patients reported lower endpoint HbA1c than paroxetine, but similar response/remission proportions and no significant fasting-glucose or BMI advantage (55). Conversely, a two-month open-label comparison involving 40 patients favoured escitalopram over agomelatine for symptoms and endpoint glycaemia (46). Together with the fluoxetine comparison (56), these small, comparator-dependent studies do not establish superiority. Reporting limitations are identified in **Supplementary Table S2**. Agomelatine requires baseline and scheduled liver testing; diabetes, obesity and fatty liver heighten the relevance of hepatic precautions (57).

Vortioxetine has a pharmacological and tolerability profile that may be useful in selected patients, but the pharmacology review is contextual evidence, not a T2D trial (58). In the direct eight-week vortioxetine-sertraline pilot, only 21 of 50 eligible participants completed treatment (59). Favourable metabolic observations in this small completer sample are exploratory. Limited average QT and weight effects in general-population trials do not establish cardiovascular protection or absence of bleeding risk (60).

Trazodone may be considered when depression coexists with insomnia, but low-dose hypnotic use is not equivalent to an adequate antidepressant regimen. The 2026 European Delphi panel describes expert opinion on selected MDD presentations, not patient-level evidence of glycaemic benefit or safety in T2D (61). Sedation, orthostasis, QT prolongation, bleeding and CYP3A4 interactions remain relevant (62). Novelty or expert preference does not establish a higher safety level than well-characterised alternatives.

### 4.3 Collaborative care and limits of comparative evidence

Collaborative care is a multicomponent service model, not a specific antidepressant treatment. Typical components include a care manager, systematic symptom and medical monitoring, adherence and self-management support, stepped adjustment and specialist case review (11, 12, 30, 63, 64). INDEPENDENT enrolled T2D with PHQ-9 scores of at least 10, not exclusively interview-confirmed MDD. Its positive composite required depression improvement and improvement in at least one cardiometabolic measure, not every component; severe hypoglycaemia was reported in eight intervention participants and none receiving usual care (12). This supports attention to diabetes-treatment safety without establishing a precise comparative harm rate. Service-level benefits cannot be assigned to antidepressant pharmacology alone.

Treatment interpretation depends on diagnostic eligibility, psychiatric need and delivery, not only on the modality label. **Table 2** identifies direct T2D findings, mixed-population evidence, bridge evidence and author interpretation. A negative or unmeasured glycaemic outcome does not mean that an otherwise useful depression treatment has failed.

**Table 2:** Comparative profile of treatment modalities for depression in type 2 diabetes.

Treatment	Depression evidence	Metabolic/cardiovascular interpretation	Potential consideration	Monitoring/limitation	References
Psychological therapy	D: benefit in selected T2D depressive-disorder trials; P: mixed-diabetes studies.	D/P: glycaemic effects vary. B/A: no established direct adverse metabolic effect; event benefit untested.	A: discuss according to diagnosis, severity, capacity and preference.	B: assess symptoms and risk. A: consider engagement and access; not sole care for acute risk.	(10, 31, 33–35)
Digital therapy	P: guided trials show benefit. D: unguided SpringboarD did not outperform active control.	D/P: consistent glycaemic benefit not established; cardiovascular events not established.	A: distinguish guided from unguided and synchronous from asynchronous delivery.	A/B: check engagement, access, privacy and escalation. Digital delivery is not itself proof of suitability.	(36–38, 94)
Exercise/behavioural care	D: symptom and remission benefit in selected T2D trials.	D: variable HbA1c findings; subgroup benefits exploratory. B: activity can alter glucose requirements.	A: discuss for medically suitable patients; consider function and preferences.	B: assess exercise suitability and hypoglycaemia risk. A: do not replace indicated psychiatric care.	(10, 39–42)
Antidepressants	B: general-MDD efficacy. D/P: small heterogeneous diabetes studies.	D/P: agent-specific glycaemic findings. B: weight, QTc, blood pressure, bleeding and interactions vary.	B/A: integrate diagnosis, severity, recurrence, prior response and preference.	B: product-specific safety and early review. D: no reliable T2D hierarchy or cardiovascular-event ranking.	(7–9, 13–18, 35, 43–45, 48, 50, 87); **Table 1**
Collaborative care	D/P: improves depression in selected service-model trials.	D: some composite or HbA1c benefit; concurrent diabetes changes prevent drug attribution.	A: consider fragmented care, multimorbidity and access to coordinated follow-up.	D/B: monitor diabetes-treatment safety, not only mood. A: clarify ownership, contact and escalation.	(11, 12, 30, 63, 64)

D, direct T2D evidence with diagnosis or symptom threshold specified; P, partially direct mixed-diabetes evidence; B, bridge studies, guidance or regulatory safety information; A, author-proposed consideration. Labels identify provenance, not certainty. Symptom-screened populations do not automatically establish efficacy in diagnosed MDD. CBT, cognitive behavioural therapy; ECG, electrocardiogram; HbA1c, glycated haemoglobin; QTc, heart-rate-corrected QT interval; T2D, type 2 diabetes.

The comparative profiles of the principal treatment modalities are summarised in **Table 2**, with evidence provenance and monitoring requirements shown explicitly.

## 5 Metabolic effects of depression treatments

### 5.1 Mechanisms, weight and glycaemic effects

A metabolic change during treatment may come from the drug, from altered appetite and activity as depression improves, or from better adherence to diabetes care. T2D studies rarely separate these pathways. They also cannot predict that the same antidepressant will change glucose in every patient. Weight, HbA1c and self-management therefore need to be measured, not inferred from an improvement in mood.

Weight effects differ between antidepressants and change with duration. Observational studies and meta-analyses, which are indirect for the present question, suggest modest differences among common agents. In a six-month target-trial emulation, bupropion was associated with less weight gain (14, 65, 66). Average estimates do not amount to a fixed ranking for T2D. They matter most when obesity, recent weight gain or disengagement related to weight makes early measurement important.

Randomised SSRI studies suggest a small average glycaemic improvement, but many are short and treat glycaemia as a secondary outcome (13). The fluoxetine placebo-controlled study involved both diabetes types and did not demonstrate a significant glycated-haemoglobin advantage (45). Sertraline recurrence-prevention data also do not establish a maintenance-phase HbA1c advantage (43). Bupropion findings are uncontrolled, and duloxetine neuropathy studies are not depression-selected T2D cohorts (48, 50). Antidepressants are not glucose-lowering treatments. Mean HbA1c changes cannot establish hypoglycaemia or hyperglycaemia incidence.

### 5.2 Other metabolic effects and time-dependent trade-offs

Data on lipids, liver enzymes and related biomarkers come mainly from broad antidepressant trials, not cohorts selected for both T2D and depression. Apparent drug differences may vary with baseline dyslipidaemia, liver disease, dose, duration and concurrent cardiometabolic treatment (15, 50). A small mean difference alone is a weak basis for choosing a drug for one patient. It becomes useful when it corresponds to a plausible individual risk and can be checked during follow-up.

Comparative rates of hypoglycaemia and hyperglycaemia are not reliably established for individual antidepressants in T2D. Published case reports include both directions of dysglycaemia, whereas a nested case-control study found no significant overall association with hypoglycaemia requiring hospital treatment (67, 68). Regulatory reports identify possible adverse events without controlled comparative incidence. Fluoxetine labelling specifically notes hypoglycaemia during treatment and hyperglycaemia after discontinuation. Appetite, insulin or sulfonylurea exposure, alcohol use and altered routines can also affect glucose safety. **Table 1** distinguishes these issues rather than equating favourable mean HbA1c findings with freedom from dysglycaemia.

Timing and mechanism are essential when metabolic results are interpreted. Acute trials capture early changes in appetite, weight, glucose and lipids. Longer studies are more likely to reveal changes in adherence, relapse and weight trajectory. Cohorts examining incident T2D or long-term weight change support surveillance, but they cannot establish causality in people with established T2D (65, 66, 69). Overall, the average metabolic changes are small, heterogeneous and hard to divide into direct drug effects and changes associated with recovery. Baseline HbA1c, alterations in diabetes treatment, and the timing of changes in mood, adherence and metabolism may tell the clinician more than antidepressant class alone.

## 6 Cardiovascular risk and safety

### 6.1 Baseline cardiovascular prognosis

Observational studies in T2D consistently identify depression as a marker of higher cardiovascular risk, not as a vascular cause known to be reversible. Smoking, inactivity, adherence, autonomic dysfunction, inflammation and the burden of diabetes may account for some of the association (3, 4, 18, 19). In Look AHEAD, associations between depressive symptoms, antidepressant use and incident cardiovascular events differed by sex. That pattern is more consistent with heterogeneity and residual confounding than with a simple drug effect (70). Cardiovascular risk factors still need to be assessed and treated directly.

An association between depression and subsequent events does not show that an antidepressant reverses that risk. Cardiovascular-event efficacy, tolerability in established cardiac disease and effects on intermediate safety markers therefore require separate interpretation.

### 6.2 Cardiovascular events and tolerability in cardiac disease

The escitalopram trial after acute coronary syndrome must be distinguished from negative heart-failure trials. In 300 patients treated for 24 weeks, long-term follow-up found fewer MACE with escitalopram than placebo (40.9% versus 53.6%; hazard ratio 0.69, 95% confidence interval 0.49-0.96; median follow-up 8.1 years) (17). This is a positive finding in a selected acute-coronary-syndrome population, not established T2D-specific cardioprotection. Mortality alone was not significantly reduced, and the result should not be generalised to all cardiovascular settings.

SADHART provides tolerability evidence for sertraline after myocardial infarction or unstable angina, rather than proof of event prevention (16). CREATE demonstrated antidepressant efficacy for citalopram in coronary disease but was not a cardiovascular-event prevention trial (71). Escitalopram did not improve death or hospitalisation in MOOD-HF, sertraline did not outperform supported placebo for depression or cardiovascular status in SADHART-CHF, and the ENRICHD psychosocial intervention did not improve event-free survival (72–74). Observational associations remain susceptible to confounding by indication, depression severity and treatment changes (70, 75, 76).

### 6.3 Intermediate safety markers, bleeding and interactions

QTc, blood pressure, pulse and orthostasis are intermediate safety outcomes. An SSRI meta-analysis found an average QTc increase of about 6 ms, with a greater signal for citalopram than several alternatives; dose, electrolytes, interactions and cardiac substrate modify individual risk (77, 78). Venlafaxine has dose-related blood-pressure effects, while several sedating or anticholinergic drugs can worsen orthostasis (79, 80). These bridge findings support risk-specific assessment, not a universal ECG schedule or a claim of haemodynamic neutrality. **Table 1** identifies agent-level precautions and their source type.

Serotonin reuptake inhibition can impair platelet aggregation. Meta-analyses have linked SSRIs to upper gastrointestinal bleeding and a relative increase in intracranial haemorrhage. The absolute risk is low, but it rises with antithrombotic or NSAID use (81–83). Most of this evidence is observational. Management should centre on bleeding history, complete medication reconciliation, gastroprotection when indicated and coordination with the clinician prescribing antithrombotic treatment. Avoiding all SSRIs may deny an effective treatment without removing the underlying bleeding risk.

For an individual patient, a drug interaction may matter more than a small average difference between antidepressants. Paroxetine and fluoxetine inhibit CYP2D6 and may raise metoprolol exposure. Fluoxetine or fluvoxamine may also reduce clopidogrel activation (84, 85). Pharmacodynamic interactions are equally relevant. Reduced food intake can destabilise glucose in patients using insulin or a sulfonylurea; serotonergic drugs add to bleeding risk; sedative or anticholinergic combinations may worsen falls and orthostasis; and several QT-active drugs can increase repolarisation risk. There is no sound basis for a class-wide claim that antidepressants increase infections with SGLT2 inhibitors or reliably augment weight loss with GLP-1 receptor agonists. These combinations should be managed by following symptoms, food and fluid intake, glucose and adverse events. Before treatment changes, review prescribed medicines, over-the-counter products, supplements, doses and organ function.

### 6.4 Boundaries of the cardiovascular evidence

Cardiovascular tolerability should not be called cardioprotection, and a QTc or blood-pressure difference is not a clinical-event estimate. Direct T2D depression trials do not establish a MACE ranking. The positive post-acute-coronary-syndrome finding remains population-specific bridge evidence, while heart-failure trials address different settings (16, 17, 72, 74). Cardiovascular risk management remains necessary regardless of the antidepressant selected.

## 7 An author-proposed conceptual checklist

### 7.1 Model scope, diagnostic triage and urgency

The checklist was assembled from recurring clinical domains in the selected literature: diagnostic and psychiatric urgency, expected symptom benefit, weight and glycaemia, cardiovascular tolerability, interactions and organ function, preferences and access. These domains can alter interpretation or feasibility; no statistical feature selection, formal stakeholder prioritisation, Delphi exercise or independent consensus process was used. No patient or multidisciplinary stakeholder co-design process is reported. The sequence is intended as a communication and reasoning checklist, not a validated decision aid, score or prescribing algorithm. Numerical weights and drug rankings were omitted because the evidence cannot justify them.

The first task is to distinguish major depressive disorder and subthreshold depressive symptoms from diabetes distress. Assessment should also consider bipolar disorder, psychosis, catatonia, severe self-neglect and whether the patient can maintain food, fluids and essential medication. Depression severity and suicide risk take priority over cardiometabolic refinement. Imminent self-harm, psychosis, catatonia or inability to meet basic needs requires urgent psychiatric assessment. If diabetes distress predominates without a depressive disorder, diabetes education and structured problem-solving may be the more appropriate response (5, 6).

### 7.2 Selecting the initial treatment modality

These modality considerations are extrapolated principally from depression guidance, not a sequence tested in T2D trials (5, 6). Psychotherapy, guided digital care or exercise may be discussed for less severe presentations when risk and capacity permit. Medication, psychotherapy or both may be considered for MDD according to severity, recurrence, previous response and preference. Severe or complex illness may require specialist and coordinated medical care. Direct T2D trials support selected interventions in their enrolled populations but do not establish a universal order (10–12, 30, 33, 39, 41, 63, 86). Distress without depressive disorder calls for diabetes-focused assessment and support rather than automatic antidepressant prescribing.

### 7.3 Metabolic, cardiovascular and interaction modifiers

Obesity or rapid weight gain can favour an option with a better weight profile, often combined with behavioural support (14, 65, 69). Poor glycaemic control is different: it is not a reason to use an antidepressant in place of diabetes treatment. The initial choice and monitoring plan should take account of appetite change, previous hypoglycaemia, insulin or sulfonylurea use and painful neuropathy. These factors create preferences that depend on the patient; they are not categorical exclusions (35, 43–45, 48, 50, 68, 77, 87, 88).

Cardiac disease, QT susceptibility, uncontrolled hypertension, orthostasis, antithrombotic treatment and interacting medicines can constrain an otherwise reasonable choice. These considerations derive mainly from cardiac studies and pharmacological safety information rather than T2D comparative trials (16, 17, 70–74, 76–82, 84, 85). Depending on drug and patient, they may justify ECG or electrolyte review, blood-pressure measurement or pharmacist-assisted interaction checks. None is a validated phenotype-specific treatment rule.

### 7.4 Shared decision-making and planned reassessment

The author-proposed checklist invites discussion of symptom relief, function, sleep, pain, weight, glucose, sexual effects, bleeding, burden and access. It does not determine a best option when constraints coexist. A jointly recorded plan can specify the target, responsible clinician and findings that would prompt adjustment. Timing is individualised according to psychiatric safety, treatment exposure and medical risk; Section 8 identifies the sources and limitations of illustrative intervals.

**Figure 3** presents this unvalidated conceptual sequence. Its usefulness and safety require testing rather than assumption.

**Figure 3:**
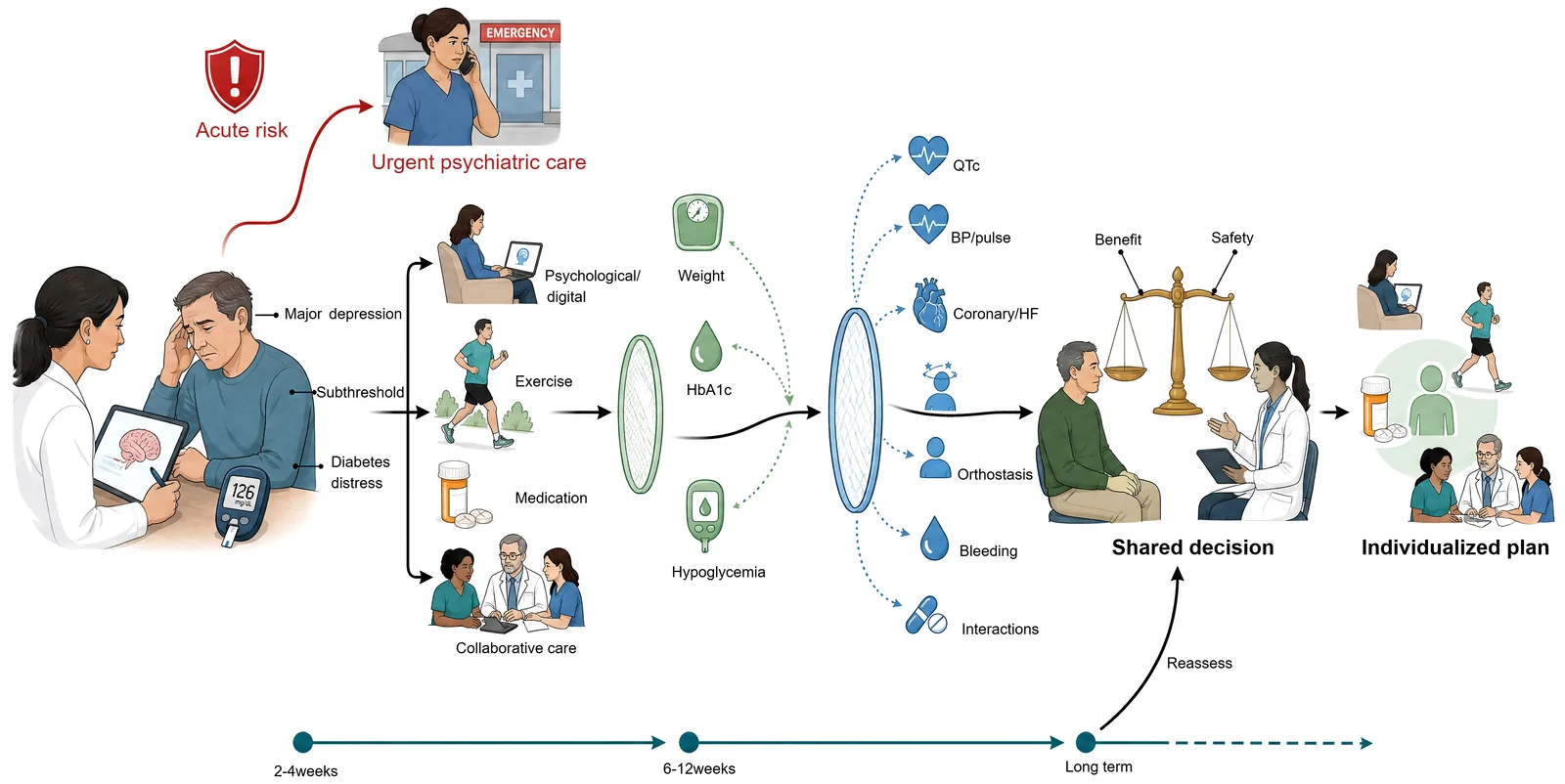
Author-proposed evidence-to-decision framework for depression treatment in type 2 diabetes. The red acute-risk pathway reflects general psychiatric safety guidance rather than a T2D treatment trial. Selected direct or partially direct diabetes studies inform the treatment options, but their populations and diagnostic criteria differ (**Tables 1**, **2**). The metabolic and cardiovascular filters draw on these findings together with extrapolated safety evidence and guidance. Their ordering, integration into a shared decision and reassessment loop are the authors’ conceptual proposal, not a validated algorithm or weighted score. The common pathway does not imply that major depression, subthreshold symptoms and diabetes distress require the same treatment; diabetes distress alone is not an indication for antidepressants. The 2-4-week, 6-12-week and long-term labels are illustrative reassessment windows, not prescribed delays. Earlier safety review follows Section 8, and acute deterioration requires immediate assessment. BP, blood pressure; HbA1c, glycated haemoglobin; HF, heart failure; QTc, heart-rate-corrected QT interval.

**Table 3** gives potential phenotype-related considerations, with provenance attached to substantive claims. It is not a prescriptive treatment matrix.

**Table 3:** Potential phenotype-related considerations for depression treatment in T2D.

Phenotype/clinical signal	Potential consideration	Main caution	Targeted monitoring	Evidence/references
Acute or severe psychiatric risk	A/B: Urgent psychiatric assessment and stabilisation	B: Do not delay effective care for metabolic optimisation or unguided treatment	B: Suicidality, intake, self-care and medication/glucose safety	B: guidance; D: sparse (5, 6)
Obesity or rapid weight gain	A/B: Psychotherapy, exercise or collaborative care; integrate prior response and weight data if prescribing	B: Weight-promoting agents; switching effective therapy for small mean differences	B: Weight, appetite, HbA1c and lipids	B (14, 15, 40, 65, 69)
Poor glycaemic control	D/P: CBT-AD and collaborative care have selected trial evidence. A: consider coordinated care when diabetes-treatment adherence is impaired.	B: Do not interpret HbA1c change as antidepressant efficacy	B/A: HbA1c, glucose and weight; distinguish diabetes-medication adherence from antidepressant use and self-management.	D/P (11–13, 33, 35, 44, 48, 63)
Recurrent hypoglycaemia	A/B: Review insulin or sulfonylurea use, meals and activity	B: Appetite or routine changes without a glucose-safety plan	B: Glucose pattern, intake and assisted episodes	B (13, 67, 68)
QT prolongation risk	A/B: Use a non-drug option or select medication after QT-drug and electrolyte review	B: Cumulative QT burden, high dose, electrolyte disturbance or bradycardia	B: Risk-triggered ECG and K/Mg; syncope or palpitations	B (15, 77, 78)
Hypertension or tachycardia	B/A: Consider options with lower expected haemodynamic burden based on non-T2D evidence; individualise titration.	B: Higher-dose venlafaxine or cumulative sympathomimetic burden	B: Seated/standing BP, pulse and palpitations	B (15, 76, 79)
Coronary disease or heart failure	A/B: Coordinate cardiac care; consider psychotherapy, collaborative care or medication with bridge-safety data	B: Do not equate mood response with cardioprotection	B: Cardiac symptoms, BP/pulse and risk-triggered ECG	B (16, 17, 71–74)
Orthostasis or falls	B/A: Consider non-drug options and lower expected orthostatic or sedating burden; individualise titration.	B: Stacked orthostatic, anticholinergic or sedating burden	B: Orthostatic vitals, dizziness, falls and gait	B (15, 19, 20, 87)
Antiplatelet or anticoagulant use	A/B: Use psychotherapy or mitigate bleeding risk if serotonergic treatment is indicated	B: NSAIDs, prior bleeding or multiple haemostasis-active drugs	B: Bleeding symptoms and medicines; CBC if indicated	B (81–83)
Polypharmacy or CYP interactions	A/B: Complete medication review; consider an interaction-light option or pharmacy input	B: Metoprolol with paroxetine/fluoxetine; clopidogrel with fluoxetine/fluvoxamine	B: Response, BP/pulse, bleeding and co-medication changes	B (80, 84, 85)
Limited digital access or literacy	A: Discuss face-to-face, telephone, community or collaborative care when digital access is unsuitable.	B: Digital-only care when access, privacy or acuity makes participation unsafe	B: Access, engagement, symptoms and suicidality	D/P (10, 36, 38, 39)

D, direct T2D finding; P, partially direct evidence; B, bridge studies, extrapolated guidance or pharmacological safety information; A, author-proposed practical consideration. These are not GRADE ratings. Rows do not establish contraindications or a treatment hierarchy. Urgent psychiatric care follows established guidance. CBT-AD, cognitive behavioural therapy for adherence and depression; CYP, cytochrome P450; ECG, electrocardiogram; HbA1c, glycated haemoglobin; NSAID, non-steroidal anti-inflammatory drug; QTc, heart-rate-corrected QT interval; T2D, type 2 diabetes.

## 8 Clinical implementation and monitoring

### 8.1 Baseline assessment and early safety review

Baseline psychiatric and medical assessment is extrapolated from depression and diabetes guidance (5, 6, 19). The authors propose recording the depressive diagnosis or screen separately from diabetes distress, together with severity, function, suicidality, prior treatment and preferences. Weight, glycaemic control, hypoglycaemia, blood pressure, organ function and medicines identify relevant constraints. ECG, electrolytes and orthostatic measurements are risk-triggered rather than universal requirements, based chiefly on pharmacological safety information and presentation (77–79, 81).

Early review follows psychiatric risk and product-specific precautions, not a T2D-validated schedule. General depression guidance provides a usual first antidepressant review within 2 weeks, or within 1 week for people aged 18–25 years or when suicide risk is a particular concern; urgent deterioration requires immediate assessment (89). Review can address activation, suicidality, tolerability, sleep, appetite, sexual effects and medication use. Glucose surveillance is relevant when insulin or sulfonylureas coincide with changing intake or activity. Blood pressure, orthostasis, ECG/electrolytes and bleeding review are selected according to drug and risk. Dose adjustments and mandatory drug-specific monitoring follow local product information, including liver monitoring for agomelatine, not a universal panel.

### 8.2 Response-guided adjustment and maintenance follow-up

Response requires serial assessment rather than a single late visit. The authors use 6–12 weeks as an illustrative acute-outcome window, not a requirement to wait before reviewing progress or changing unsafe or ineffective care. Depression guidance informs assessment of dose, exposure, adherence, function and partial response (6, 89). Optimisation, psychotherapy augmentation, switching or specialist review depends on the course. Important hypoglycaemia, hypertension, syncope, bleeding or suspected arrhythmia requires prompt medical evaluation; no single review-derived numerical threshold is proposed for all patients.

Continuation treatment helps consolidate remission. Maintenance treatment is more strongly indicated after recurrent, severe or chronic depression. There is no T2D-specific duration trial that supports a different schedule, so depression guidelines, relapse-prevention evidence and individual recurrence risk should determine dose and duration (6, 43, 66, 88). Follow-up intervals should reflect baseline risk and recent treatment changes. The measures may include relapse, function, adherence, weight, HbA1c, lipids, blood pressure, falls, bleeding and drug interactions. Antidepressants should be tapered rather than stopped abruptly. Decisions about tapering depend on previous episodes, residual symptoms, adverse burden and patient preference. If cardiometabolic control worsens, the medical problem needs active treatment and the overall benefit should be reconsidered before the change is attributed to the antidepressant.

**Figure 4** presents an author-proposed illustration of reassessment. Its legend distinguishes the illustrative windows from guidance-derived safety review; the diagram is not a T2D-specific protocol.

**Figure 4:**
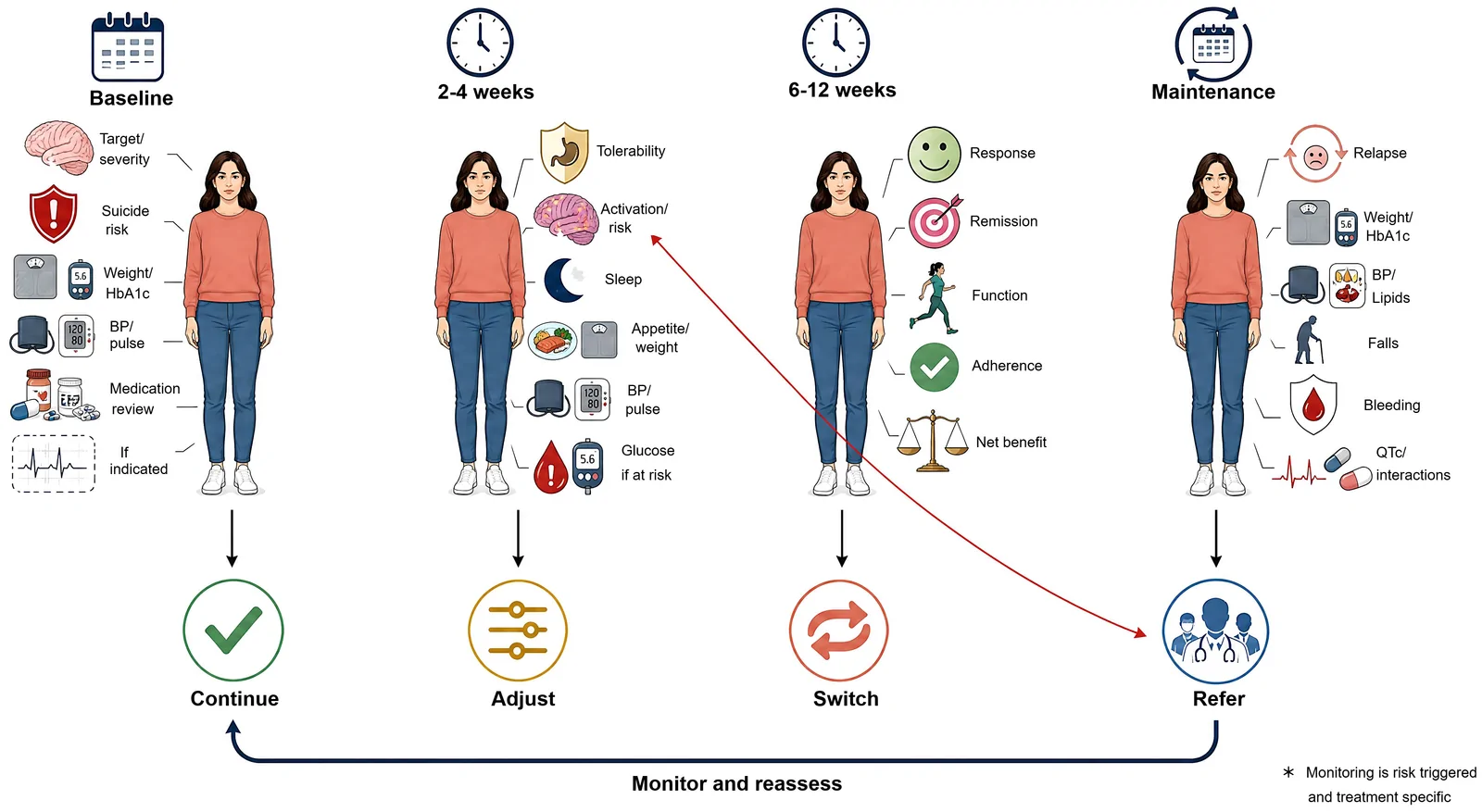
Author-proposed illustration of risk-triggered monitoring and reassessment in type 2 diabetes. The baseline, 2-4-week, 6-12-week and maintenance columns organise potential review domains; this sequence has not been validated as a T2D-specific follow-up protocol. Psychiatric and metabolic domains are informed by selected diabetes studies, whereas ECG/QTc, bleeding, interactions and drug-specific surveillance rely mainly on extrapolated guidance and safety information (**Tables 1**, **2**; **Supplementary Tables S2**, **S3**). The 2-4-week column must not be read as the timing of the first antidepressant safety review. NICE guidance usually advises review within 2 weeks, or within 1 week for people aged 18–25 years or when suicide risk is a particular concern (89); acute deterioration requires immediate assessment. ECG and other investigations are risk-triggered, and product-specific monitoring takes precedence. Continue, adjust, switch and refer are alternative actions available whenever clinically indicated, not mandatory actions tied to the columns above them. Response, tolerability, preferences and individual risk determine reassessment. BP, blood pressure; HbA1c, glycated haemoglobin; QTc, heart-rate-corrected QT interval.

### 8.3 Collaborative care and special populations

Monitoring is useful only if responsibility and escalation routes are clear. Primary care or diabetes teams can coordinate routine measurements. Psychiatry and psychology are better placed to address diagnostic uncertainty, treatment resistance and complex risk, while cardiology may be needed for unstable disease or difficult interactions. Collaborative-care studies support systematic measurement and adjustment of treatment, but cardiometabolic improvement still requires active management of the medical targets (11, 12, 30, 63, 64). A practical handover identifies the target symptom, the responsible clinician, the date of the next review and the finding that should prompt adjustment or referral.

This review concerns adults and does not provide treatment advice for adolescents, pregnancy or breastfeeding. Those populations need separate evidence and specialist guidance. Within adult T2D, frailty and older age increase the relevance of falls, orthostasis, hyponatraemia and polypharmacy, while renal and hepatic impairment affect exposure and permitted dosing. Painful neuropathy may influence a choice such as duloxetine, but analgesic efficacy does not establish superior antidepressant efficacy. **Table 1** records relevant constraints; local prescribing information and individual assessment remain necessary (5, 6, 20, 50).

### 8.4 Access, real-world effectiveness and health economics

Real-world effectiveness depends on access, continuity, clinical ownership and cost, as well as on efficacy. Observational studies include patients with multimorbidity and can follow longer exposure or uncommon events. Their causal interpretation is weakened by indication, depression severity, adherence and treatment switching (14, 65, 70, 75, 76, 90). Collaborative care may be good value in some systems, including the Indian INDEPENDENT programme, but the result depends on staffing, programme intensity, local prices and which outcomes are counted (91). Digital care can reduce travel and scheduling barriers. It can also widen inequity when device access, literacy, privacy, engagement or a safe escalation route is missing. Reporting implementation separately from efficacy helps distinguish failure of delivery from failure of the treatment itself.

## 9 Evidence gaps and research priorities

### 9.1 Direct evidence, reporting and framework validation

The clearest evidence gap is the lack of adequately powered, long-term active-comparator trials in T2D that are stratified by phenotype. Existing pharmacotherapy studies are small and brief, and cardiovascular trials in related populations do not answer the T2D-specific question (8, 16–18, 43, 70–76, 82, 84, 85). A useful pragmatic trial would follow participants for at least 12 months. It would stratify baseline HbA1c and cardiovascular risk, record treatment changes and adherence, and report psychiatric, metabolic, safety and patient-prioritised outcomes in the same cohort.

Future comparisons would be easier to interpret if psychiatric response and remission, function, diabetes-medication adherence, antidepressant adherence, weight, HbA1c, hypoglycaemia and important adverse events were reported together. Digital and collaborative-care studies also need descriptions of guidance, contact intensity, engagement, escalation and costs. **Table 4** is an author-proposed minimum reporting framework with illustrative windows. Neither its outcomes nor intervals have undergone stakeholder consensus, Delphi development or validation as a core outcome set (21, 22, 27, 42, 63, 64).

**Table 4:** Author-proposed minimum reporting framework and illustrative measurement windows.

Outcome	Proposed measure	Acute (0–12 weeks)	Continuation (3–12 months)	Long term (>12 months)	References
Depression response/remission	A: Validated scale; continuous change, response, remission and suicidality	A: Baseline and serial progress; 6-12-week acute outcome. Earlier safety review follows guidance (89).	A: 3, 6 and 12 months	A: Annually and at relapse	(7–10, 31, 35, 43)
Function/quality of life	A: Validated functional and diabetes-specific or generic quality-of-life measure	A: Baseline; 6–12 weeks	A: 6 and 12 months	A: Annually or after a major event	(12, 33, 38, 42, 44)
Adherence/self-management	A: Separate antidepressant adherence, diabetes-medication adherence and broader self-management; specify measure.	A: Baseline; each contact	A: 3, 6 and 12 months	A: Annual persistence	(11, 12, 29, 33, 63)
Weight/waist	A: Standardised weight; waist if prespecified	A: Baseline; 4 weeks if high risk; 12 weeks	A: 3, 6 and 12 months	A: Annual trajectory	(14, 15, 65, 66)
HbA1c/hypoglycaemia	A: Laboratory HbA1c; biochemical and severe hypoglycaemia	A: Baseline; about 12 weeks; surveillance throughout	A: HbA1c every 3–6 months; cumulative events	A: Annual; severe events	(11–13, 28, 44, 48, 67, 68)
BP/pulse/QTc	A: Seated BP/pulse; standing BP and ECG/QTc when indicated	A/B: Baseline and after relevant treatment changes; ECG is risk-triggered, not universal.	A: Repeat as required by the research question, drug and clinical risk.	A: Reassess with symptoms or interactions; no universal ECG interval.	(12, 15, 77–79)
Bleeding	A: Major or clinically relevant bleeding; site and severity	A: Event surveillance throughout; early review follows drug and patient risk.	A: Cumulative through 12 months	A: Continuous time-to-event	(81–83)
CV events/mortality	A: Adjudicated MI, stroke, HF admission and CV/all-cause death	A: Capture all events	A: Cumulative events and admissions	A: Primary time-to-event assessment	(3, 16–19, 70–74)
Relapse/discontinuation	A: Time to relapse; reason-specific discontinuation, switching or rescue	A: Early discontinuation and switching	A: 3, 6 and 12 months	A: Time-to-event beyond 12 months	(7, 8, 43, 88)
Preference/implementation	A: Preference, reach, uptake, acceptability, burden, equity and escalation	A: Baseline preference; engagement throughout	A: 3, 6 and 12 months	A: Sustainability, equity and adoption	(5, 6, 36–39, 42)

Author-proposed minimum reporting framework, not a consensus-derived core outcome set or clinical follow-up protocol. All windows are illustrative design suggestions (A); citations identify relevant outcomes, not validation of the schedule. Event surveillance spans follow-up; ECG and other tests depend on the research question and safety indications. BP, blood pressure; ECG, electrocardiogram; HbA1c, glycated haemoglobin; QTc, heart-rate-corrected QT interval; T2D, type 2 diabetes.

Validation of the checklist would begin with patient and multidisciplinary clinician input to identify missing domains and unclear language. A feasibility study could measure completion time, missing items, acceptability and agreement between assessors on domain identification. A subsequent prospective comparison would assess decision quality, depression, function, treatment burden and adverse events alongside glycaemic outcomes. Inter-rater agreement alone would not establish benefit or safety, and stakeholder refinement would not constitute clinical validation.

Health-record studies can be informative about longer-term outcomes if the causal question, eligibility, treatment assignment, follow-up and time-varying confounding are specified before analysis. They complement pragmatic randomisation; they do not replace it. Three lines of work would be particularly useful: active-comparator T2D trials with at least 12 months of follow-up, mediation studies measuring depression, adherence and HbA1c together, and implementation or economic studies of collaborative and digital care. Existing studies of weight, mortality and cardiovascular events show why indication, adherence and depression severity must be handled explicitly (14, 65, 70, 75, 76, 91).

### 9.2 Underrepresented phenotypes, equity and emerging therapies

Older adults and people with kidney disease, autonomic neuropathy, recurrent severe hypoglycaemia or advanced complications are inadequately represented in many adult T2D depression trials. Future work should prespecify justified subgroup questions and report organ function, frailty and exposure. Pregnancy, breastfeeding and adolescence lie outside this adult synthesis and require distinct evidence rather than extrapolated rankings (5, 6, 20, 50).

New metabolic treatments may affect observed mood through weight, glucose, behaviour or care, but cardiometabolic benefit alone does not establish an antidepressant indication. Observational GLP-1 receptor agonist studies require attention to comparator choice, treated depression and confounding (90, 92, 93). The short agomelatine comparisons and attrition-limited vortioxetine pilot require replication; trazodone consensus is not a substitute for T2D trials. Comparative T2D evidence for esketamine remains insufficient to establish its cardiometabolic place.

### 9.3 Limitations of this review

Purposive selection creates a risk of omitted or preferentially cited evidence. Original database-specific search histories, restrictions, screening counts and complete exclusion records cannot be reconstructed from the materials available for revision. Selection and extraction were not consistently independently duplicated. Revision-stage source verification, a cited-report inventory and qualitative appraisals improve traceability but cannot retrospectively make the review systematic. Some appraisals rely on abstracts; funding or conflicts remain unverified where disclosures were unavailable. No formal *de novo* risk-of-bias or GRADE assessment was conducted. Heterogeneous diagnoses, co-interventions, outcomes and follow-up prevent a quantitative benefit-harm hierarchy. Much safety evidence is indirect or observational. The checklist and monitoring illustrations are author interpretations without stakeholder consensus or prospective validation.

## 10 Conclusion

Depression treatment in T2D requires attention to psychiatric need and drug-specific metabolic, cardiovascular and interaction constraints. Direct evidence remains limited, and diagnosed depression, elevated scores and diabetes distress should not be conflated. Individual agents and nonpharmacological programmes have useful findings, but these do not establish a universal hierarchy, reliable comparative dysglycaemia rates or T2D-specific cardiovascular protection.

The checklist makes these distinctions visible without converting them into a prescribing rule. Its utility remains a hypothesis. Reproducible evidence synthesis, longer active-comparator studies and prospective evaluation involving patients and clinicians are needed before stronger recommendations can be made.

## Glossary

AMSTAR 2: A Measurement Tool to Assess Systematic Reviews 2
BMI: body mass index
BP: blood pressure
CBC: complete blood count
CBT: cognitive behavioural therapy
CBT-AD: cognitive behavioural therapy for adherence and depression
CENTRAL: Cochrane Central Register of Controlled Trials
CV: cardiovascular
CYP: cytochrome P450
CYP2D6: cytochrome P450 2D6
DAD: Diabetes and Depression study
ECG: electrocardiogram
GLP-1: glucagon-like peptide-1
GRADE: Grading of Recommendations Assessment, Development and Evaluation
HbA1c: glycated haemoglobin
HF: heart failure
MACE: major adverse cardiovascular events
MEDLINE: Medical Literature Analysis and Retrieval System Online
MI: myocardial infarction
NSAID: non-steroidal anti-inflammatory drug
PRISMA-ScR: Preferred Reporting Items for Systematic Reviews and Meta-Analyses extension for Scoping Reviews
PsycINFO: APA PsycINFO database
QT: QT interval
QTc: heart-rate-corrected QT interval
RoB 2: revised Cochrane risk-of-bias tool for randomized trials
ROBINS-E: Risk Of Bias In Non-randomized Studies of Exposures
ROBINS-I: Risk Of Bias In Non-randomized Studies of Interventions
SADHART-CHF: Sertraline Against Depression and Heart Disease in Chronic Heart Failure
SGLT2: sodium-glucose cotransporter 2
SNRI: serotonin-noradrenaline reuptake inhibitor
SSRI: selective serotonin reuptake inhibitor
SWiM: Synthesis Without Meta-analysis
T2D: type 2 diabetes
MDD: major depressive disorder
