# Supplementary material for: Choosing depression treatment in type 2 diabetes: integrating psychiatric efficacy, metabolic effects, and cardiovascular risk. A narrative review
Source: Front Psychiatry. 2026 Oct 19;17:1976656. doi: 10.3389/fpsyt.2026.1976656 (PMC13725209; doi:10.3389/fpsyt.2026.1976656)
Supplement: Supplementary file 1 [file Table1.docx]

Supplementary Material

Choosing Depression Treatment in Type 2 Diabetes: Integrating Psychiatric Efficacy, Metabolic Effects, and Cardiovascular Risk. A Narrative Review

Manuscript 1976656 | Targeted source verification during revision, 8 September 2026

# Table S1 Literature selection and verification record

| **Item** | **Record and limitation** |
| --- | --- |
| Review identity | Narrative review using purposively selected literature. No systematic or scoping-review claim. |
| Original search cutoff | 27 July 2026 was stated in the submitted manuscript. Original search histories were not available for verification during revision. |
| Original databases | The submitted manuscript named PubMed/MEDLINE, Embase, PsycINFO, CENTRAL, Web of Science and Scopus. Complete strategies and execution dates cannot be verified from the supplied materials. This is not a newly reconstructed six-database search. |
| Retained example query | ("Diabetes Mellitus, Type 2"[Mesh] OR "type 2 diabetes"[tiab] OR T2DM[tiab]) AND ("Depressive Disorder"[Mesh] OR depression[tiab] OR depressive[tiab]) AND (antidepressant*[tiab] OR psychotherap*[tiab] OR exercise[tiab] OR "collaborative care"[tiab]). This submitted example does not represent the separate safety, drug-specific or implementation domains. |
| Restrictions and selection records | Original language, publication-status, conference and grey-literature restrictions, deduplication counts, exclusion reasons and disagreement resolution could not be verified. No retrospective PRISMA counts or dual-reviewer process are asserted. |
| Revision-stage verification | Targeted checks of cited and reviewer-suggested reports via PubMed/Europe PMC indexed records, DOI/Crossref or publisher records, available full texts, NICE guidance and regulatory labels. Public record checks were conducted on 8 September 2026. These checks are not an exhaustive literature update. |
| Access limitations | Full text was used where available; individual Table S2 records state retrieval scope. An abstract-derived judgement is not a full-text risk-of-bias appraisal. Unverified funding/conflicts are recorded as unverified, not absent. |
| Report linkage | Lustman 2007 bupropion and Sayuk 2011 sexual-function analysis share a cohort. Program ACTIVE II acute and follow-up reports share a trial. Existing reviews and networks overlap with individual studies and with one another. |
| Inventory scope | The main manuscript reference list contains the complete cited bibliography; the supplementary tables selectively document source verification and study appraisal. Table S2 selectively appraises studies material to comparisons. Neither table proves complete study identification. |

# Table S2 Selected study-level qualitative appraisal

These records make population, outcome and interpretation limits visible. They are not formal RoB 2, ROBINS-I, AMSTAR 2 or GRADE assessments and do not replace independent full-text appraisal. The table retains study-level source links and limitations in a single continuous layout.

| **Study / report** | **Katon 2010, TEAMcare \| Revised ref 11 (original 11)** |
| --- | --- |
| Design and sample | Single-blind RCT, n=214 |
| Diabetes population | Poorly controlled diabetes, coronary heart disease, or both; not exclusively T2D |
| Depression ascertainment | Coexisting depression; exact diagnostic/symptom entry criterion not verified from abstract |
| Intervention and comparator | Nurse-led, medically supervised collaborative management of depression, glycaemia, blood pressure and lipids versus usual care |
| Follow-up | 12 months |
| Psychiatric outcomes | Greater improvement in SCL-20 scores |
| Cardiometabolic outcomes | Joint outcome favored intervention; between-group improvements were 0.58 percentage points HbA1c, 6.9 mg/dL LDL and 5.1 mmHg systolic BP |
| Main limitations | Complex unblinded care package and simultaneous changes in diabetes, antihypertensive and antidepressant treatment prevent attribution to depression treatment alone; not a cardiovascular-event efficacy trial |
| Directness | Partially direct mixed chronic-disease service-model evidence |
| Funding and conflicts | NIMH funding verified; PubMed reports no other relevant conflict, which is not a complete itemization of declared relationships |
| Retrieval scope | Abstract and PubMed metadata |
| Source records | <https://pubmed.ncbi.nlm.nih.gov/21190455/>; <https://doi.org/10.1056/NEJMoa1003955> |
| **Study / report** | Ali 2020, INDEPENDENT \| Revised ref 12 (original 12) |
| Design and sample | Open-label pragmatic RCT, n=404; 378 completed |
| Diabetes population | T2D with poorly controlled HbA1c, BP or LDL; four clinics in India |
| Depression ascertainment | Elevated depressive symptoms, PHQ-9 >=10; do not relabel as interview-confirmed MDD |
| Intervention and comparator | Care coordinators, self-management support, electronic decision support and specialist case review versus usual care |
| Follow-up | 12-month intervention plus 12-month postintervention follow-up |
| Psychiatric outcomes | Primary composite required at least 50% SCL-20 reduction |
| Cardiometabolic outcomes | Composite also required improvement in HbA1c, SBP or LDL; 71.6% versus 57.4% met outcome at 24 months; severe hypoglycaemia 8 versus 0 |
| Main limitations | Open-label multicomponent intervention; composite cannot establish benefit on every component; most individual secondary outcomes did not differ; rare event counts not definitive safety comparisons |
| Directness | Direct T2D symptom-threshold evidence for a service package, not drug efficacy |
| Funding and conflicts | NIMH grant verified; complete COI statement not verified |
| Retrieval scope | Abstract and PubMed metadata |
| Source records | <https://pubmed.ncbi.nlm.nih.gov/32809002/>; <https://doi.org/10.1001/jama.2020.11747> |
| **Study / report** | Kim 2018, escitalopram after acute coronary syndrome \| Revised ref 17 (original 17) |
| Design and sample | Double-blind placebo-controlled RCT, n=300 |
| Diabetes population | Recent acute coronary syndrome; not a T2D-specific trial |
| Depression ascertainment | Depression; exact interview-defined categories require full-text confirmation |
| Intervention and comparator | Escitalopram 5-20 mg/day versus placebo for 24 weeks |
| Follow-up | Median 8.1-year cardiac follow-up; complete follow-up reported |
| Psychiatric outcomes | Long-term report focused on cardiac events, not a new antidepressant efficacy comparison |
| Cardiometabolic outcomes | MACE 40.9% versus 53.6%; HR 0.69, 95% CI 0.49-0.96; mortality alone not significantly different |
| Main limitations | Single-center ACS trial; limited precision; short randomized exposure compared with long follow-up; cannot establish T2D-specific cardioprotection or class-wide benefit |
| Directness | Bridge cardiovascular-event efficacy evidence; supportive result must not be described as null |
| Funding and conflicts | MRC support indexed; full funding and COI statement not verified |
| Retrieval scope | Abstract and PubMed metadata |
| Source records | <https://pubmed.ncbi.nlm.nih.gov/30043065/>; <https://doi.org/10.1001/jama.2018.9422> |
| **Study / report** | Katon 2004, Pathways \| Revised ref 30 (original 30) |
| Design and sample | RCT, n=329; blinded outcome assessments |
| Diabetes population | Diabetes, type composition not verified in accessible abstract |
| Depression ascertainment | Major depression and/or dysthymia |
| Intervention and comparator | Case management supporting antidepressant treatment or problem-solving therapy versus usual care |
| Follow-up | 12 months |
| Psychiatric outcomes | Depression severity, perceived improvement and adequacy of antidepressant treatment improved |
| Cardiometabolic outcomes | No between-group HbA1c difference |
| Main limitations | Service package cannot be blinded; glycaemic null result cautions against assuming mood improvement alone improves glycaemia |
| Directness | Diabetes depression service evidence; do not label exclusively T2D without confirming population |
| Funding and conflicts | NIMH grants verified; complete COI statement not verified |
| Retrieval scope | Abstract and PubMed metadata |
| Source records | <https://pubmed.ncbi.nlm.nih.gov/15466678/>; <https://doi.org/10.1001/archpsyc.61.10.1042> |
| **Study / report** | Newby 2017, guided internet CBT \| Revised ref 36 (original 33) |
| Design and sample | RCT, 106 randomized; 91 completed baseline and entered analysis |
| Diabetes population | Mixed T1D/T2D, with a high proportion of T1D |
| Depression ascertainment | MDD confirmed by telephone MINI; very severe symptoms/high suicide risk excluded |
| Intervention and comparator | Six online CBT lessons with email/telephone therapist support versus treatment as usual |
| Follow-up | 10 weeks; intervention-only follow-up at 3 months |
| Psychiatric outcomes | Posttreatment PHQ-9 and diabetes-distress improvements; PHQ-9 Hedges g=0.78 |
| Cardiometabolic outcomes | No significant difference in self-reported HbA1c |
| Main limitations | Postrandomization exclusions, attrition, self-reported HbA1c, no control follow-up, and unblinded follow-up diagnostic interviews |
| Directness | Partially direct mixed-diabetes diagnosed-MDD evidence; not evidence for unguided digital care |
| Funding and conflicts | No specific project grant; institutional support and author fellowships acknowledged; no conflicts declared |
| Retrieval scope | Abstract and full text |
| Source records | <https://pubmed.ncbi.nlm.nih.gov/28506956/>; <https://pmc.ncbi.nlm.nih.gov/articles/PMC5447827/> |
| **Study / report** | Clarke 2019, SpringboarD \| Revised ref 38 (original 34) |
| Design and sample | RCT, 780 randomized as reported; published arm counts contain inconsistencies |
| Diabetes population | T2D; online self-selected sample |
| Depression ascertainment | PHQ-2 >=2; PHQ-9 >19 excluded; symptom-based recruitment, not diagnosed MDD |
| Intervention and comparator | Fully automated self-guided myCompass CBT versus active online Healthy Lifestyles control |
| Follow-up | 8 weeks plus 4-week tailing-off; 3-month assessment |
| Psychiatric outcomes | No specific superiority for functioning, depression or diabetes distress |
| Cardiometabolic outcomes | No demonstrated glycaemic efficacy; principal reported secondary outcomes concerned self-management |
| Main limitations | Low engagement, substantial attrition, mild baseline symptoms/floor effects and self-selected sample; do not pool interpretation with guided MDD trials |
| Directness | Direct T2D elevated-symptom evidence, not an MDD-treatment trial |
| Funding and conflicts | NHMRC grant 1083116; Australian Government supported program development; no conflicts declared |
| Retrieval scope | Abstract and full text |
| Source records | <https://pubmed.ncbi.nlm.nih.gov/31115345/>; <https://pmc.ncbi.nlm.nih.gov/articles/PMC6548532/> |
| **Study / report** | Petrak 2015, DAD \| Revised ref 35 (original 36) |
| Design and sample | Single-blind RCT, n=251; responders entered continuation phase |
| Diabetes population | Mixed T1D/T2D with poor glycaemic control |
| Depression ascertainment | SCID-confirmed major depression |
| Intervention and comparator | Diabetes-specific group CBT versus sertraline; bibliotherapy versus continued sertraline after acute response |
| Follow-up | 12-week acute phase followed by 1-year continuation |
| Psychiatric outcomes | Both improved; sertraline showed an advantage in depression-score reduction during longer follow-up |
| Cardiometabolic outcomes | No significant between-group HbA1c difference |
| Main limitations | Only acute responders entered continuation; unequal continuation interventions; mixed diabetes limits T2D-specific inference |
| Directness | Partially direct diabetes MDD evidence; not proof of metabolic equivalence of all antidepressants and CBT |
| Funding and conflicts | Not verified in accessible abstract/metadata |
| Retrieval scope | Abstract and PubMed metadata |
| Source records | <https://pubmed.ncbi.nlm.nih.gov/25690005/>; <https://doi.org/10.2337/dc14-1599> |
| **Study / report** | Angermann 2016, MOOD-HF \| Revised ref 72 (original 53) |
| Design and sample | Double-blind placebo-controlled RCT, n=372 exposed; early stopping |
| Diabetes population | Chronic systolic heart failure, LVEF <45%; not T2D-specific |
| Depression ascertainment | Diagnostic interview after PHQ-9 screening |
| Intervention and comparator | Escitalopram 10-20 mg/day versus placebo, both with optimal heart-failure therapy |
| Follow-up | Planned 24 months; median participation approximately 18.5 months |
| Psychiatric outcomes | No significant depression-score advantage at 12 weeks |
| Cardiometabolic outcomes | Death/hospitalization HR 0.99, 95% CI 0.76-1.27; safety parameters comparable |
| Main limitations | Early stopping and imprecision; selected cardiac population; no demonstrated T2D-specific effect |
| Directness | Bridge heart-failure event and tolerability evidence; distinct from ACS trial original ref 17 [revised 17] |
| Funding and conflicts | Not verified in accessible abstract/metadata |
| Retrieval scope | Abstract and PubMed metadata |
| Source records | <https://pubmed.ncbi.nlm.nih.gov/27367876/>; <https://doi.org/10.1001/jama.2016.7635> |
| **Study / report** | Lesperance 2007, CREATE \| Revised ref 71 (original 55) |
| Design and sample | 2x2 factorial RCT, n=284 |
| Diabetes population | Coronary artery disease; not T2D-specific |
| Depression ascertainment | DSM-IV major depression >=4 weeks; HAM-D-24 >=20 |
| Intervention and comparator | Citalopram 20-40 mg/day versus placebo; IPT plus clinical management versus clinical management alone |
| Follow-up | 12 weeks |
| Psychiatric outcomes | Citalopram improved HAM-D, response and remission; IPT did not add benefit over clinical management |
| Cardiometabolic outcomes | Not a trial establishing prevention of cardiovascular events or T2D glycaemic benefit |
| Main limitations | Short duration; psychotherapy cannot be blinded; intensive clinical-management comparator; historical doses must not override current cardiac safety restrictions |
| Directness | Bridge antidepressant efficacy in CAD; not direct T2D evidence |
| Funding and conflicts | Not verified in accessible abstract/metadata |
| Retrieval scope | Abstract and PubMed metadata |
| Source records | <https://pubmed.ncbi.nlm.nih.gov/17244833/>; <https://doi.org/10.1001/jama.297.4.367> |
| **Study / report** | Berkman 2003, ENRICHD \| Revised ref 73 (original 58) |
| Design and sample | RCT, n=2481 |
| Diabetes population | Recent MI; not T2D-specific |
| Depression ascertainment | Major/minor depression and/or low perceived social support; some participants did not have depression |
| Intervention and comparator | CBT-based psychosocial intervention with SSRI when indicated versus usual medical care |
| Follow-up | Intervention over 6 months; mean cardiac follow-up 29 months |
| Psychiatric outcomes | Greater improvement in depression and social support |
| Cardiometabolic outcomes | No improvement in death/recurrent-MI-free survival: 75.8% versus 75.9% |
| Main limitations | Unblinded complex intervention, heterogeneous eligibility and substantial usual-care improvement; drug use was not independently randomized |
| Directness | Bridge psychosocial cardiac-event evidence; not a specific antidepressant cardioprotection trial |
| Funding and conflicts | NHLBI contracts verified; complete COI statement not verified |
| Retrieval scope | Abstract and PubMed metadata |
| Source records | <https://pubmed.ncbi.nlm.nih.gov/12813116/>; <https://doi.org/10.1001/jama.289.23.3106> |
| **Study / report** | O'Connor 2010, SADHART-CHF \| Revised ref 74 (original 61) |
| Design and sample | Double-blind placebo-controlled RCT, n=469 |
| Diabetes population | Heart failure, LVEF <=45%; not T2D-specific |
| Depression ascertainment | DSM-IV current MDD |
| Intervention and comparator | Sertraline 50-200 mg/day versus placebo; nurse-facilitated support in both arms |
| Follow-up | 12 weeks |
| Psychiatric outcomes | No greater HAM-D reduction with sertraline |
| Cardiometabolic outcomes | No improvement in composite cardiovascular status; short-term tolerability acceptable in selected HF patients |
| Main limitations | Short duration, intensive support in both groups, selected exclusions; cannot establish long-term or T2D-specific cardiovascular safety |
| Directness | Bridge HF tolerability and short-term efficacy evidence |
| Funding and conflicts | NIMH grant verified; complete COI statement not verified |
| Retrieval scope | Abstract and PubMed metadata |
| Source records | <https://pubmed.ncbi.nlm.nih.gov/20723799/>; <https://doi.org/10.1016/j.jacc.2010.03.068> |
| **Study / report** | Safren 2014, CBT-AD \| Revised ref 33 (original 62) |
| Design and sample | RCT, n=87 |
| Diabetes population | T2D with suboptimal glycaemic control |
| Depression ascertainment | MINI/DSM-IV: 66 current major depressive episodes, 11 dysthymia, 10 MDD in partial remission on antidepressants |
| Intervention and comparator | 9-11 CBT-AD sessions plus enhanced usual care versus enhanced usual care alone, including diabetes-adherence counseling |
| Follow-up | 4-month treatment; follow-up through 12 months |
| Psychiatric outcomes | MADRS improved at 4 months; between-group depression differences not sustained across later follow-up |
| Cardiometabolic outcomes | Better diabetes-medication and glucose-monitoring adherence; HbA1c 0.72 percentage points lower at 4 months, 0.63 across subsequent follow-up |
| Main limitations | Small trial, unblinded therapy, additional contact, heterogeneous depressive diagnoses; benefits cannot be assigned to mood treatment alone |
| Directness | Direct T2D evidence for combined depression/adherence intervention, not pure depression CBT |
| Funding and conflicts | NIH funding; donated LifeScan glucometers/strips; author book royalties and consulting relationships disclosed |
| Retrieval scope | Abstract and full text |
| Source records | <https://pubmed.ncbi.nlm.nih.gov/24170758/>; <https://pmc.ncbi.nlm.nih.gov/articles/PMC3931377/> |
| **Study / report** | Piette 2011, telephone CBT plus walking \| Revised ref 39 (original 63) |
| Design and sample | RCT, n=291 |
| Diabetes population | T2D |
| Depression ascertainment | Elevated depressive symptoms, BDI >=14; not necessarily diagnosed MDD |
| Intervention and comparator | Nurse-delivered telephone CBT with pedometer walking versus control |
| Follow-up | 12 weekly sessions and 9 monthly boosters; 12-month outcomes |
| Psychiatric outcomes | Depression improved; reported remission 58% versus 39% |
| Cardiometabolic outcomes | No HbA1c benefit; systolic BP approximately 4.26 mmHg lower; more steps/day |
| Main limitations | Combined CBT/exercise intervention; relatively good baseline HbA1c limits room for change; telephone contact is synchronous guided care |
| Directness | Direct T2D symptom-threshold evidence, not unguided digital treatment |
| Funding and conflicts | NIH grants verified; complete COI statement not verified |
| Retrieval scope | Abstract and PubMed metadata |
| Source records | <https://pubmed.ncbi.nlm.nih.gov/21478777/>; <https://doi.org/10.1097/MLR.0b013e318215d0c9> |
| **Study / report** | Saha 2024, Program ACTIVE II follow-up \| Revised ref 41 (original 64) |
| Design and sample | Follow-up of original ref 71 [revised 42] RCT; n=87 at 6 months, n=75 at 12 months |
| Diabetes population | T2D; same trial as original ref 71 [revised 42], not an independent cohort |
| Depression ascertainment | MDD at original trial entry |
| Intervention and comparator | CBT, exercise, combined CBT/exercise, usual care |
| Follow-up | 6 and 12 months |
| Psychiatric outcomes | All groups showed clinically significant symptom improvement; some psychosocial differences favored combination treatment |
| Cardiometabolic outcomes | HbA1c benefit for combination treatment in baseline HbA1c >=7% subgroup; explicitly preliminary |
| Main limitations | Attrition from original n=140 and small subgroup; avoid double-counting this follow-up as an independent trial |
| Directness | Direct T2D MDD evidence, exploratory subgroup metabolic finding |
| Funding and conflicts | NIDDK funding; de Groot consultant to Mediflix; Mather employee of Eli Lilly disclosed |
| Retrieval scope | Abstract and PubMed funding/COI statement |
| Source records | <https://pubmed.ncbi.nlm.nih.gov/38266570/>; <https://doi.org/10.1016/j.jdiacomp.2023.108666> |
| **Study / report** | Bogner 2012, integrated management \| Revised ref 63 (original 65) |
| Design and sample | RCT, n=180 |
| Diabetes population | T2D prescribed diabetes and depression pharmacotherapy |
| Depression ascertainment | Treatment-based entry; diagnostic interview or threshold not verified; do not assume current MDD in every participant |
| Intervention and comparator | Care-manager education, adherence monitoring and guideline-based recommendations versus usual care |
| Follow-up | 12 weeks |
| Psychiatric outcomes | PHQ-9 <5 remission 58.7% versus 30.7% |
| Cardiometabolic outcomes | HbA1c <7% achieved by 60.9% versus 35.7%; monitored diabetes and antidepressant medication adherence separately |
| Main limitations | Short unblinded service intervention; treatment-based selection and multiple co-interventions limit drug-level conclusions |
| Directness | Direct T2D treated-depression service evidence; case-definition boundary requires caution |
| Funding and conflicts | NIMH grants verified; complete COI statement not verified |
| Retrieval scope | Abstract and PubMed metadata |
| Source records | <https://pubmed.ncbi.nlm.nih.gov/22230826/>; <https://doi.org/10.1370/afm.1344> |
| **Study / report** | Nobis 2015, GET.ON Mood Enhancer Diabetes \| Revised ref 37 (original 70) |
| Design and sample | RCT, n=260 |
| Diabetes population | Mixed T1D/T2D |
| Depression ascertainment | Elevated symptoms, CES-D >=23; not an MDD-only sample |
| Intervention and comparator | Guided web self-help with mobile support versus brief unguided online psychoeducation |
| Follow-up | 2 months |
| Psychiatric outcomes | Depressive-symptom reduction d=0.89 and diabetes-distress reduction d=0.58, intention-to-treat |
| Cardiometabolic outcomes | HbA1c or CV benefit not established by the accessible report |
| Main limitations | Short duration, self-report outcomes and unequal guidance/intensity; cannot isolate digital platform from support effects |
| Directness | Partially direct mixed-diabetes symptom evidence; distress is an outcome, not distress-only eligibility |
| Funding and conflicts | Not verified in accessible abstract/metadata |
| Retrieval scope | Abstract and PubMed metadata |
| Source records | <https://pubmed.ncbi.nlm.nih.gov/25710923/>; <https://doi.org/10.2337/dc14-1728> |
| **Study / report** | de Groot 2019, Program ACTIVE II \| Revised ref 42 (original 71) |
| Design and sample | 2x2 factorial RCT, n=140 across four groups |
| Diabetes population | T2D for at least 1 year |
| Depression ascertainment | DSM-IV-TR MDD; remission assessed by interviewers blinded to assignment |
| Intervention and comparator | CBT, exercise, combined CBT/exercise versus usual care |
| Follow-up | 12 weeks; later follow-up in original ref 64 [revised 41] |
| Psychiatric outcomes | Adjusted odds of full MDD remission approximately 5.0-6.8 times usual care |
| Cardiometabolic outcomes | Combined treatment improved HbA1c; large benefit in HbA1c >=7% subgroup was exploratory post hoc |
| Main limitations | Small four-arm study, unblinded intervention, demographic concentration; subgroup findings are hypothesis-generating |
| Directness | Direct T2D diagnosed-MDD evidence; exercise alone not established as a universal replacement for indicated pharmacotherapy |
| Funding and conflicts | NIDDK grants verified; complete original-report COI statement not verified |
| Retrieval scope | Abstract and PubMed metadata |
| Source records | <https://pubmed.ncbi.nlm.nih.gov/31221693/>; <https://doi.org/10.2337/dc18-2400> |
| **Study / report** | Lustman 1998, CBT with diabetes education \| Revised ref 34 (original 74) |
| Design and sample | RCT, n=51; reported remission denominators 20-22 per arm |
| Diabetes population | T2D |
| Depression ascertainment | Major depression; remission defined by BDI <=9 |
| Intervention and comparator | 10-week individual CBT versus no specific antidepressant treatment; diabetes education in both arms |
| Follow-up | 10 weeks and 6-month posttreatment follow-up |
| Psychiatric outcomes | Higher remission with CBT after treatment and at follow-up |
| Cardiometabolic outcomes | No immediate glycaemic difference; lower glycosylated haemoglobin at 6 months |
| Main limitations | Small referral-center sample, reduced analyzed sample, unblinded psychotherapy and symptom-based remission definition; imprecise generalizability |
| Directness | Direct T2D MDD evidence, limited sample |
| Funding and conflicts | NIMH grant verified; complete COI statement not verified |
| Retrieval scope | Abstract and PubMed metadata |
| Source records | <https://pubmed.ncbi.nlm.nih.gov/9786808/>; <https://doi.org/10.7326/0003-4819-129-8-199810150-00005> |
| **Study / report** | Atlantis 2014, collaborative-care meta-analysis \| Revised ref 64 (original 76) |
| Design and sample | Systematic review of 7 RCTs; depression n=1895, HbA1c n=1556 |
| Diabetes population | Diabetes populations across included trials; not uniformly T2D-only |
| Depression ascertainment | Clinically relevant depression with heterogeneous trial definitions |
| Intervention and comparator | Multidisciplinary collaborative care versus control care |
| Follow-up | Short-to-medium term; varied by trial |
| Psychiatric outcomes | Pooled depression SMD -0.32, 95% CI -0.53 to -0.11 |
| Cardiometabolic outcomes | HbA1c weighted mean difference -0.33 percentage points, 95% CI -0.66 to -0.00; remission did not predict glycaemic benefit across studies |
| Main limitations | High heterogeneity (depression I2=79%, HbA1c 72.9%), heterogeneous service components and mostly US trials; overlaps with primary studies above and must not be counted independently |
| Directness | Partially direct synthesis; depression and metabolic effects need separate interpretation |
| Funding and conflicts | No specific grant; Atlantis disclosed speaking honoraria from Eli Lilly Australia |
| Retrieval scope | Abstract and full-text review |
| Source records | <https://pubmed.ncbi.nlm.nih.gov/24727428/>; <https://pmc.ncbi.nlm.nih.gov/articles/PMC3987739/> |
| **Study / report** | Srisurapanont 2022, pharmacotherapy network \| Revised ref 8 (original 8) |
| Design and sample | Systematic review/network meta-analysis; 12 RCTs, n=792 |
| Diabetes population | T2D and depression |
| Depression ascertainment | Constituent study definitions vary; not a uniform MDD diagnostic cohort |
| Intervention and comparator | Eight antidepressants; placebo/no treatment and active comparisons |
| Follow-up | 8-24 weeks |
| Psychiatric outcomes | Some favourable network estimates; sparse direct comparisons |
| Cardiometabolic outcomes | HbA1c network signals for some drugs; no reliable clinical-event comparison |
| Main limitations | Small network, short trials, heterogeneous comparators; no new AMSTAR 2 or GRADE judgement inferred |
| Directness | Direct T2D synthesis with overlapping constituent trials; not evidence for duloxetine or bupropion |
| Funding and conflicts | Not verified from retrieved abstract |
| Retrieval scope | Abstract and metadata |
| Source records | <https://pubmed.ncbi.nlm.nih.gov/35691471/> |
| **Study / report** | Lustman 2006, sertraline recurrence prevention \| Revised ref 43 (original 37) |
| Design and sample | Double-blind placebo-controlled RCT, n=152 after open-label recovery |
| Diabetes population | Mixed diabetes; 82.9% T2D |
| Depression ascertainment | Recovered MDD; DSM-IV recurrence outcome |
| Intervention and comparator | Continued sertraline versus placebo after sertraline induction |
| Follow-up | Up to 52 weeks after induction |
| Psychiatric outcomes | Lower recurrence hazard, HR 0.51 (95% CI 0.31-0.85) |
| Cardiometabolic outcomes | HbA1c did not differ during randomised maintenance |
| Main limitations | Responder-enriched maintenance; mixed diabetes; cannot infer initial-drug superiority |
| Directness | Partially direct recurrence-prevention evidence |
| Funding and conflicts | Not verified from retrieved abstract |
| Retrieval scope | Abstract and metadata |
| Source records | <https://pubmed.ncbi.nlm.nih.gov/16651509/> |
| **Study / report** | Lustman 2000, fluoxetine \| Revised ref 45 (original 38) |
| Design and sample | Double-blind placebo-controlled RCT, n=60 |
| Diabetes population | 26 T1D, 34 T2D |
| Depression ascertainment | Major depression; BDI/HAMD measured; exact diagnostic interview not verified |
| Intervention and comparator | Fluoxetine up to 40 mg/day versus placebo |
| Follow-up | 8 weeks |
| Psychiatric outcomes | Depressive symptoms improved more with fluoxetine |
| Cardiometabolic outcomes | Glycated-haemoglobin difference not significant |
| Main limitations | Small mixed-diabetes trial, short duration; no robust harm-event assessment |
| Directness | Partially direct |
| Funding and conflicts | Not verified from retrieved abstract |
| Retrieval scope | Abstract and metadata |
| Source records | <https://pubmed.ncbi.nlm.nih.gov/10834419/> |
| **Study / report** | Lustman 2007 / Sayuk 2011, bupropion cohort \| Revised ref 48 (original 39) |
| Design and sample | Open-label parent cohort n=93; sexual-function subset n=90 |
| Diabetes population | T2D with MDD |
| Depression ascertainment | DSM-IV-TR MDD; BDI measures; exact interview instrument not verified |
| Intervention and comparator | Bupropion; no control group. original ref 87 [revised 49] is a secondary analysis |
| Follow-up | 10-week acute phase; remitters continued 24 weeks |
| Psychiatric outcomes | Depression improved; sexual functioning improved in secondary analysis |
| Cardiometabolic outcomes | BMI and HbA1c declined; self-care improved during treatment |
| Main limitations | Uncontrolled, 75/93 acute completers; remitter-enriched maintenance; reports share cohort |
| Directness | Direct population, observational treatment association rather than causal effect |
| Funding and conflicts | Secondary report declares no relevant conflicts; parent funding not verified |
| Retrieval scope | Parent abstract; secondary full text |
| Source records | <https://pubmed.ncbi.nlm.nih.gov/17327305/>; <https://pmc.ncbi.nlm.nih.gov/articles/PMC3024343/> |
| **Study / report** | Hardy 2007, duloxetine neuropathic-pain analysis \| Revised ref 50 (original 41) |
| Design and sample | Pooled 3 trials, n=1024; extension n=867 |
| Diabetes population | Diabetes with peripheral neuropathic pain, not depression-selected |
| Depression ascertainment | No depressive-disorder entry criterion established |
| Intervention and comparator | Duloxetine versus placebo; long-term routine-care comparison |
| Follow-up | 12-week controlled phase and 52-week extension |
| Psychiatric outcomes | Not an antidepressant-efficacy study |
| Cardiometabolic outcomes | Modest fasting-glucose increases; long-term HbA1c rise 0.52% versus 0.19%; early weight loss |
| Main limitations | Indication indirectness; pooled/extension designs and different long-term comparator |
| Directness | Bridge safety, not direct T2D-depression efficacy |
| Funding and conflicts | Specific funding and complete conflicts not verified from retrieved abstract |
| Retrieval scope | Abstract and metadata |
| Source records | <https://pubmed.ncbi.nlm.nih.gov/17192327/> |
| **Study / report** | Tovilla-Zárate 2019, vortioxetine-sertraline pilot \| Revised ref 59 (original 78) |
| Design and sample | Single-blind pilot with reported randomisation; 50 eligible, 21 completed |
| Diabetes population | T2D, HbA1c at least 7.5%; ages 18-60 |
| Depression ascertainment | Psychiatrist-confirmed DSM-5 depression; HDRS at least 14 |
| Intervention and comparator | Vortioxetine 10 mg/day versus sertraline 75 mg/day |
| Follow-up | 8 weeks |
| Psychiatric outcomes | Symptoms improved in both arms |
| Cardiometabolic outcomes | Exploratory favourable metabolic changes with vortioxetine among completers |
| Main limitations | 58% noncompletion; small final sample; single blinding; unstable estimates |
| Directness | Direct T2D depressive-disorder pilot |
| Funding and conflicts | Not verified from retrieved disclosure material |
| Retrieval scope | Abstract and accessible publisher full text; no granular effect extraction |
| Source records | <https://pubmed.ncbi.nlm.nih.gov/31930057/>; <https://atm.amegroups.org/article/view/30925/html> |
| **Study / report** | Karaiskos 2013, agomelatine-sertraline \| Revised ref 54 (original 81) |
| Design and sample | Open-label comparative pilot, reported random allocation, n=40 |
| Diabetes population | T2D with suboptimal control |
| Depression ascertainment | Depression reported; exact diagnostic threshold not available in abstract |
| Intervention and comparator | Agomelatine versus sertraline |
| Follow-up | 4 months |
| Psychiatric outcomes | Symptoms and self-care favoured agomelatine |
| Cardiometabolic outcomes | Lower endpoint HbA1c; no significant final weight or fasting-glucose effect |
| Main limitations | Design terminology inconsistent, small unblinded study, no placebo |
| Directness | Direct population, limited diagnostic detail |
| Funding and conflicts | Not verified from retrieved abstract |
| Retrieval scope | Abstract and metadata |
| Source records | <https://pubmed.ncbi.nlm.nih.gov/23409693/> |
| **Study / report** | Kang 2015, agomelatine-paroxetine \| Revised ref 55 (original 82) |
| Design and sample | Reported double-blind RCT, n=116 |
| Diabetes population | T2D |
| Depression ascertainment | DSM-IV assessment; HDRS at least 17 |
| Intervention and comparator | Agomelatine versus paroxetine; active comparator |
| Follow-up | 12 weeks |
| Psychiatric outcomes | Response/remission proportions did not significantly differ |
| Cardiometabolic outcomes | Lower endpoint HbA1c with agomelatine; fasting glucose/BMI not significantly different |
| Main limitations | No placebo; unusual dose reporting; apparently reversed remission cutoff; inconsistent claims across abstract/results |
| Directness | Direct T2D depressive-disorder study with reporting limitations |
| Funding and conflicts | No financial support; authors declared no conflicts in full text |
| Retrieval scope | Full text |
| Source records | <https://pmc.ncbi.nlm.nih.gov/articles/PMC4455852/> |
| **Study / report** | Kumar 2015, agomelatine-escitalopram \| Revised ref 46 (original 83) |
| Design and sample | Randomised open-label study, n=40 |
| Diabetes population | T2D |
| Depression ascertainment | ICD-10 depression; GHQ-Hindi greater than 15, HDRS at least 14 |
| Intervention and comparator | Agomelatine 25 mg/day versus escitalopram 10 mg/day |
| Follow-up | 2 months |
| Psychiatric outcomes | Endpoint symptom scores favoured escitalopram |
| Cardiometabolic outcomes | Month-2 HbA1c/fasting glucose favoured escitalopram; endpoint differences not baseline-adjusted effects |
| Main limitations | Small, unblinded; baseline imbalance; abstract/table discrepancy for month-1 significance |
| Directness | Direct T2D depressive-disorder comparison |
| Funding and conflicts | No explicit funding/COI statement located in retrieved publisher text |
| Retrieval scope | Publisher full text; incomplete Crossref metadata not used to reject identity |
| Source records | <https://doi.org/10.13040/IJPSR.0975-8232.6(10).4304-09> |
| **Study / report** | Young 2026, trazodone Delphi \| Revised ref 61 (original 85) |
| Design and sample | Two-round Delphi; 32 experts, 8 countries; no patient cohort |
| Diabetes population | Expert opinion concerning MDD presentations |
| Depression ascertainment | No patient-level diagnostic eligibility |
| Intervention and comparator | Expert agreement; no intervention/control patient comparison |
| Follow-up | Consensus rounds, not clinical follow-up |
| Psychiatric outcomes | 68/91 statements reached strong consensus |
| Cardiometabolic outcomes | No measured T2D HbA1c or cardiovascular outcomes |
| Main limitations | Panel of regular trazodone users; industry sponsorship; opinions cannot estimate benefit or adverse-event incidence |
| Directness | Contextual expert consensus |
| Funding and conflicts | Angelini sponsored meeting and writing support; multiple industry relationships disclosed |
| Retrieval scope | Full text |
| Source records | <https://pmc.ncbi.nlm.nih.gov/articles/PMC13122520/> |
| **Study / report** | Crucitti 2010, duloxetine outside DPNP \| Revised ref 51 (original 86) |
| Design and sample | Pooled short-term studies; duloxetine n=1563, placebo n=1098 |
| Diabetes population | GAD, fibromyalgia, low-back pain and a separate long-term MDD study |
| Depression ascertainment | Not a T2D-plus-depression cohort |
| Intervention and comparator | Duloxetine versus placebo; separate uncontrolled extension |
| Follow-up | 9-27 weeks; extensions 41/52 weeks |
| Psychiatric outcomes | Not the principal question of the glycaemic analysis |
| Cardiometabolic outcomes | No consistent placebo-adjusted HbA1c/fasting-glucose change; small within-group extension HbA1c rise |
| Main limitations | Indication indirectness; uncontrolled and controlled estimates must not be conflated |
| Directness | Bridge safety; cannot establish T2D glycaemic neutrality |
| Funding and conflicts | Specific sponsor/COI statement not verified; industry affiliations noted but not equated with study sponsorship |
| Retrieval scope | Abstract and metadata |
| Source records | <https://pubmed.ncbi.nlm.nih.gov/20874076/> |
| **Study / report** | Che 2018, agomelatine versus fluoxetine \| Revised ref 56 (original 79) |
| Design and sample | Single-blind RCT, n=84; fluoxetine 40 and agomelatine 44 |
| Diabetes population | T2D, HbA1c >7%; aged 18-80 years |
| Depression ascertainment | HDRS-17 >=17 and HARS >7; symptom-threshold sample, not verified interview-confirmed MDD |
| Intervention and comparator | Agomelatine 25-50 mg/day versus fluoxetine 30-40 mg/day |
| Follow-up | 12 weeks |
| Psychiatric outcomes | HDRS improved in both groups with no significant treatment-group main effect; response/remission proportions similar; greater HARS improvement with agomelatine |
| Cardiometabolic outcomes | Lower adjusted endpoint HbA1c with agomelatine; no significant BMI or fasting-glucose advantage; no cardiovascular-event efficacy assessment |
| Main limitations | Small single-centre study; participants unblinded, raters blinded; short follow-up and symptom-selected sample. A nonsignificant difference is not proof of equivalence. Multiple outcomes and limited safety power constrain inference. |
| Directness | Direct T2D symptom-threshold evidence; not proof of superiority in diagnosed MDD or long-term cardiometabolic safety |
| Funding and conflicts | Not verified from the retrieved sections |
| Retrieval scope | Available full-text eligibility, design, outcome and analysis sections plus indexed abstract |
| Source records | <https://pubmed.ncbi.nlm.nih.gov/29942131/>; <https://europepmc.org/articles/PMC6005323> |

# Table S3 Operational provenance rules

| **Category** | **Criterion and example** | **Consequence for interpretation** |
| --- | --- | --- |
| D Direct | T2D with diagnosed depressive disorder or an explicit validated symptom threshold. CBT-AD and INDEPENDENT enrol different diagnostic populations. | State ascertainment and measured outcome. A symptom-screened result is not automatically an MDD estimate. Directness does not remove study bias. |
| P Partially direct | Mixed/unspecified diabetes with no T2D-specific estimate. Fluoxetine and sertraline recurrence-prevention studies. | Report the mixed population and available T2D proportion. Do not imply a T2D-specific comparison. |
| B Bridge efficacy | General MDD trial or synthesis without a diabetes-specific analysis. | Supports general psychiatric efficacy, not a diabetes-specific metabolic advantage or ranking. |
| B Bridge safety or guidance | Cardiac trials, clinical guidelines and regulatory labels. Post-ACS escitalopram, heart-failure trials, NICE NG222 and product information. | Restrict inference to the setting and outcome. Tolerability, QTc, blood pressure and MACE are distinct. Guidance extrapolation is not T2D trial evidence. |
| C Contextual | Mechanisms, case reports, spontaneous reports or expert consensus, including trazodone Delphi. | Can identify plausibility, signals and research questions. No comparative incidence or clinical efficacy follows. |
| A Author proposal | Conceptual checklist and illustrative reporting/review windows. | Explicitly unvalidated, without formal consensus or clinical decision-rule status. |

Categories are claim-specific and may overlap within a report. A major mismatch is a population, indication or outcome difference that prevents direct interpretation of the target claim, such as pain without depression, no T2D-specific estimate, or surrogate markers substituted for events. Categories were applied descriptively during revision; no independent assignment, disagreement adjudication or inter-rater reliability is claimed.
